# Serine Hydroxymethyltransferase ShrA (PA2444) Controls Rugose Small-Colony Variant Formation in *Pseudomonas aeruginosa*

**DOI:** 10.3389/fmicb.2018.00315

**Published:** 2018-02-27

**Authors:** Mingming Pu, Lili Sheng, Sooyeon Song, Ting Gong, Thomas K. Wood

**Affiliations:** Department of Chemical Engineering, Pennsylvania State University, University Park, PA, United States

**Keywords:** rugose, small colony variants, serine hydroxymethyltransferase, *Pseudomonas aeruginosa*, biofilm formation

## Abstract

*Pseudomonas aeruginosa* causes many biofilm infections, and the rugose small-colony variants (RSCVs) of this bacterium are important for infection. We found here that inactivation of PA2444, which we determined to be a serine hydroxymethyltransferase (SHMT), leads to the RSCV phenotype of *P. aeruginosa* PA14. In addition, loss of PA2444 increases biofilm formation by two orders of magnitude, increases exopolysaccharide by 45-fold, and abolishes swarming. The RSCV phenotype is related to higher cyclic diguanylate concentrations due to increased activity of the Wsp chemosensory system, including diguanylate cyclase WspR. By characterizing the PA2444 enzyme *in vitro*, we determined the physiological function of PA2444 protein by relating it to *S*-adenosylmethionine (SAM) concentrations and methylation of a membrane bound methyl-accepting chemotaxis protein WspA. A whole transcriptome analysis also revealed PA2444 is related to the redox state of the cells, and the altered redox state was demonstrated by an increase in the intracellular NADH/NAD^+^ ratio. Hence, we provide a mechanism for how an enzyme of central metabolism controls the community behavior of the bacterium, and suggest the PA2444 protein should be named ShrA for serine hydroxymethyltransferase related to rugose colony formation.

## Introduction

*Pseudomonas aeruginosa* is an opportunistic pathogen that is responsible for many biofilm infections including those associated with ventilator-associated pneumonia, urinary and peritoneal dialysis catheters, bacterial keratitis, otitis externa, lungs (Macé et al., 2008), and burn wounds (Gjødsbøl et al., 2006). Persistence of this bacterium is linked to its ability to form biofilms (Ryder et al., 2007) and rugose small-colony variants (RSCVs) (Drenkard and Ausubel, 2002); for example, approximately 30% of the antibiotic-resistant colonies of *P. aeruginosa* clinical isolate PA14 were RSCVs (Drenkard and Ausubel, 2002). RSCVs have a wrinkled colony morphology, and these cells have elevated aggregation, attachment, and exopolysaccharide (EPS) production (Starkey et al., 2009). Previously, our lab discovered that tyrosine phosphatase TpbA controls the RSCV phenotype in *P. aeruginosa* through diguanylate cyclase (DGC) TpbB (Ueda and Wood, 2009; Pu and Wood, 2010). Our original findings were verified by an independent group which corroborated that TpbB is important for persistence related to cystic fibrosis through RSCVs (Malone et al., 2010). RSCVs are important for infection but their regulation is poorly characterized so it is an important challenge to identify the molecular mechanisms behind the formation of these variants (Häussler, 2010).

**Figure 1:**
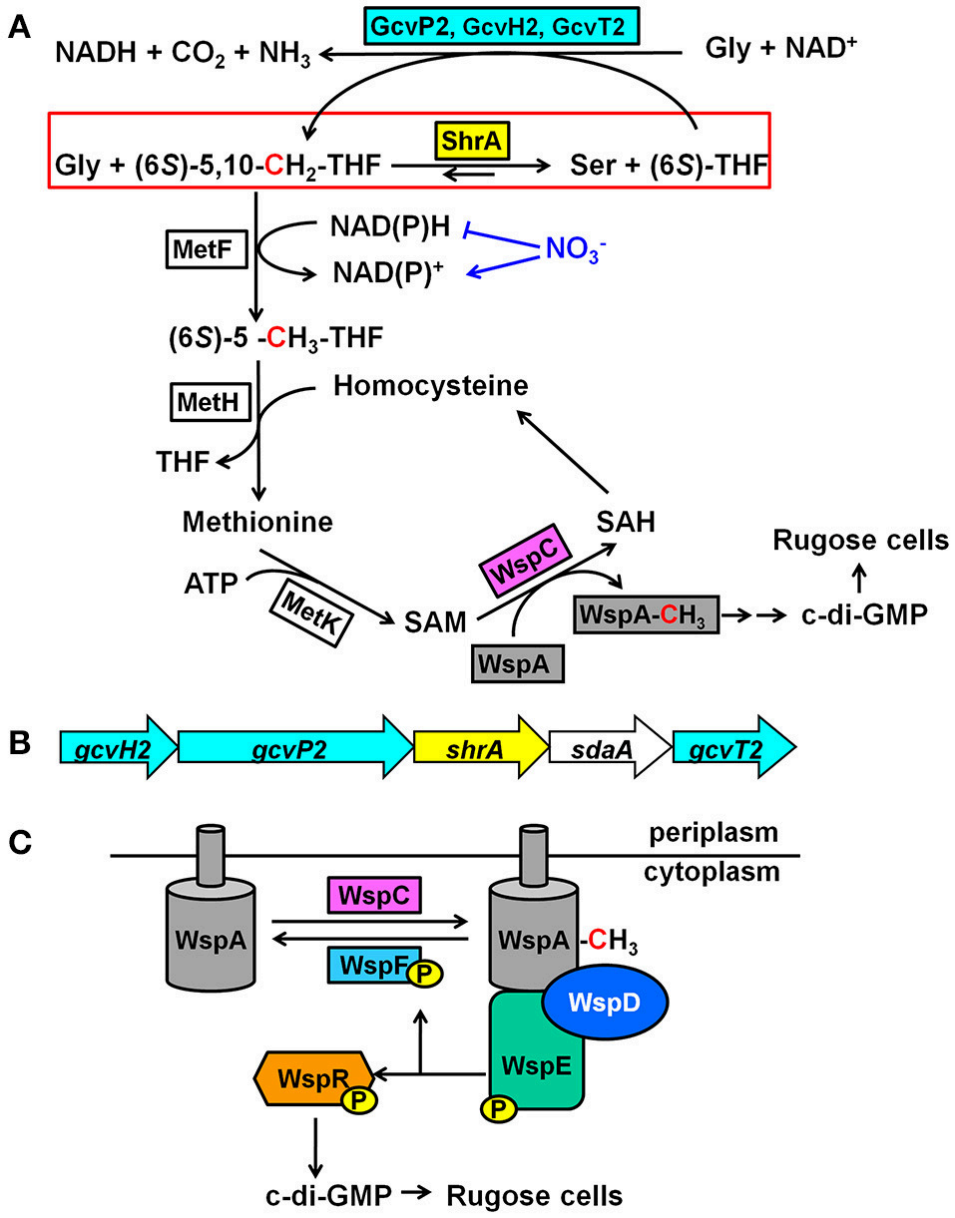
Mechanism of how ShrA controls c-di-GMP concentrations. **(A)** Proposed mechanism of how ShrA controls rugose morphology. The red box highlights the reaction scheme for SHMT ShrA. **(B)** Organization of the genes encoding ShrA and the glycine cleavage system. **(C)** Scheme for the Wsp chemosensory system. For the metabolites, THF is tetrahydrofolate, SAM is *S*-adenosylmethionine, SAH is *S*-adenosylhomocysteine and c-di-GMP is cyclic diguanylate; for the proteins, Gcv is the glycine cleavage system, MetF is a 5,10-methylenetetrahydrofolate reductase, MetH is a methionine synthase, MetK is a methionine methyltransferase, WspA is a membrane bound methyl-accepting chemotaxis protein, WspC is a methyltransferase, WspF is a methylesterase, WspD is a scaffold protein, WspE is a histidine kinase, and WspR is a diguanylate cyclase.

Serine hydroxymethyltransferase (SHMT) is a pyridoxal 5′-phosphate (PLP)-dependent enzyme which has been extensively studied from different species as it is one of the few PLP-dependent enzymes that can be found in all living organisms (Florio et al., 2010). SHMT catalyzes the reversible conversion of glycine and (6*S*)-5,10-methylene-tetrahydrofolate [(6*S*)-5,10-CH_2_-THF] to l-serine and (6*S*)-tetrahydrofolate [(6*S*)-THF] (Schirch and Szebenyi, 2005; Figure 1A); SHMT is utilized by the cell for generating one-carbon fragments for the synthesis of diverse metabolites such as quorum-sensing (QS) signals, nucleotides, methionine, thymidylate, and choline (Rao et al., 2003). Bacterial SHMTs from *Escherichia coli* (Scarsdale et al., 2000), *Bacillus* species (Bhatt et al., 2010) and *Mycobacterium tuberculosis* (Chaturvedi and Bhakuni, 2003) have been characterized, and usually these studies focused on enzyme mechanism and sometimes the role of SHMT in the regulation of central metabolism. However, a SHMT from *Pseudomonas* sp. has not been characterized and the physiological function is not understood.

In this study, we demonstrate that the SHMT ShrA (serine hydroxymethyltransferase related to rugose formation) from *P. aeruginosa*, which is encoded by *shrA* (PA2444, previously annotated as *glyA2* based on bioinformatics), controls rugose colony morphology by regulating the second messenger cyclic diguanylate (c-di-GMP) through the Wsp system. We characterized the physiological function of this reversible enzyme by assaying *in vitro* enzyme activity and found that ShrA catalyzes the reaction to form Ser and (6*S*)-THF faster than it catalyzes the reaction to form Gly and (6*S*)-5,10-CH_2_-THF. When inactivated, the loss of ShrA should cause a build-up of (6*S*)-5,10-CH_2_-THF which would drive methylation of WspA which results in an increase c-di-GMP levels and biofilm formation. Our results provide one of the first links between central metabolism and cell community behavior.

## Results

### Inactivation of ShrA leads to RSCV morphology

**Figure 2:**
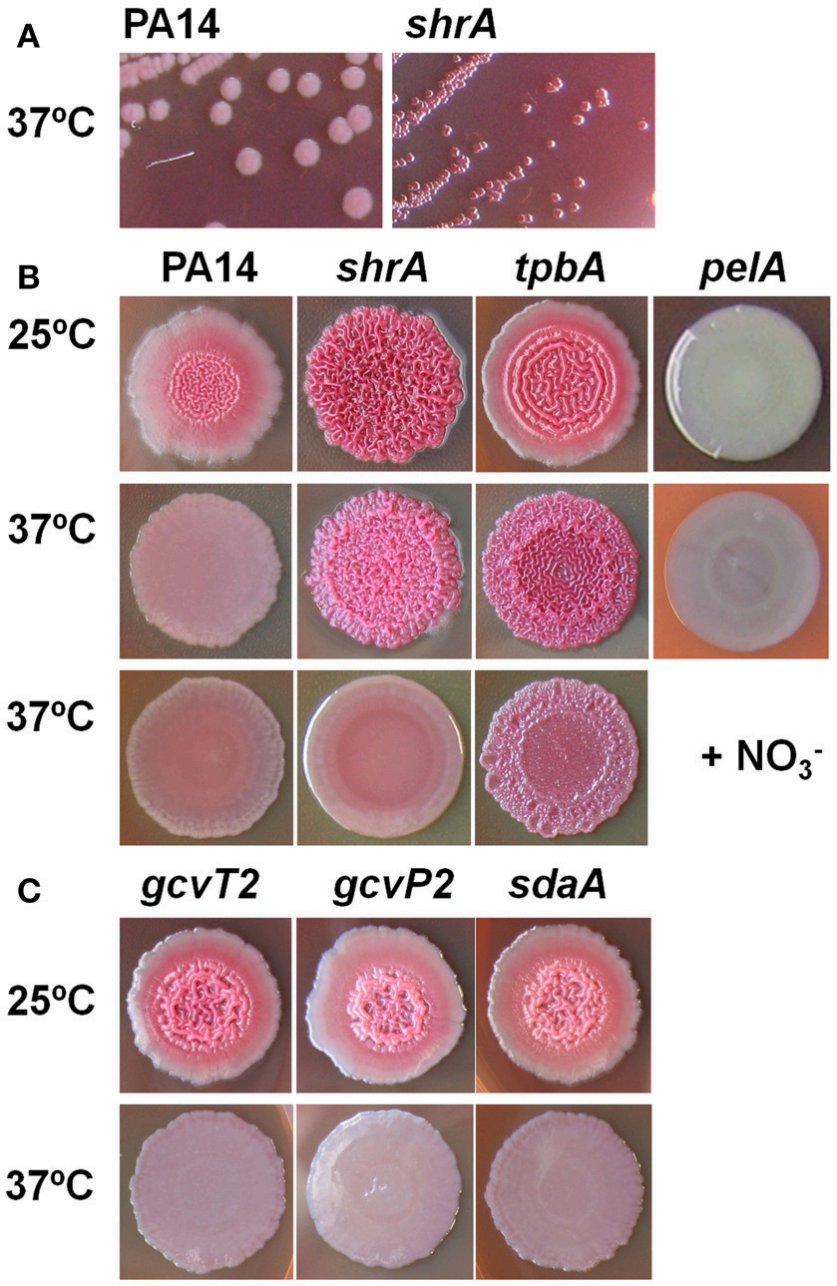
Inactivation of *shrA* leads to RSCV morphology. **(A)** Colony morphology on Congo-red plates after 2 days at 37°C showing the smaller size of the *shrA* mutant. **(B)** Colony morphology after 6 days at 25°C, after 3 days at 37°C, and after 3 days at 37°C with 30 mM KNO_3_. **(C)** Morphology of *gcvP2, gcvT2* and *sdaA* mutants in the *shrA* operon.

Previously, by screening 5,850 transposon mutants for altered biofilm formation, we identified 137 transposon mutants of *P. aeruginosa* PA14 with over 3-fold enhanced biofilm formation (Ueda et al., 2009). We then tested the colony morphology on Congo-red plates for 28 strains with the highest biofilm formation and identified a strain, the *shrA* mutant, with the RSCV phenotype in addition to the previously identified *tpbA* mutant (Ueda and Wood, 2009; Figure 2A). These colonies were smaller in size than the PA14 wild-type strain (Figure 2A) and developed a distinctive red and wrinkled morphology at both 37° and 25°C within 1~2 days whereas the wild-type was smooth and non-wrinkled at 37°C and only slightly rough at 25°C (Figure 2B). Therefore, the *shrA* mutation converts wild-type cells into the RSCV phenotype.

### Inactivation of ShrA increases biofilm formation

**Figure 3:**
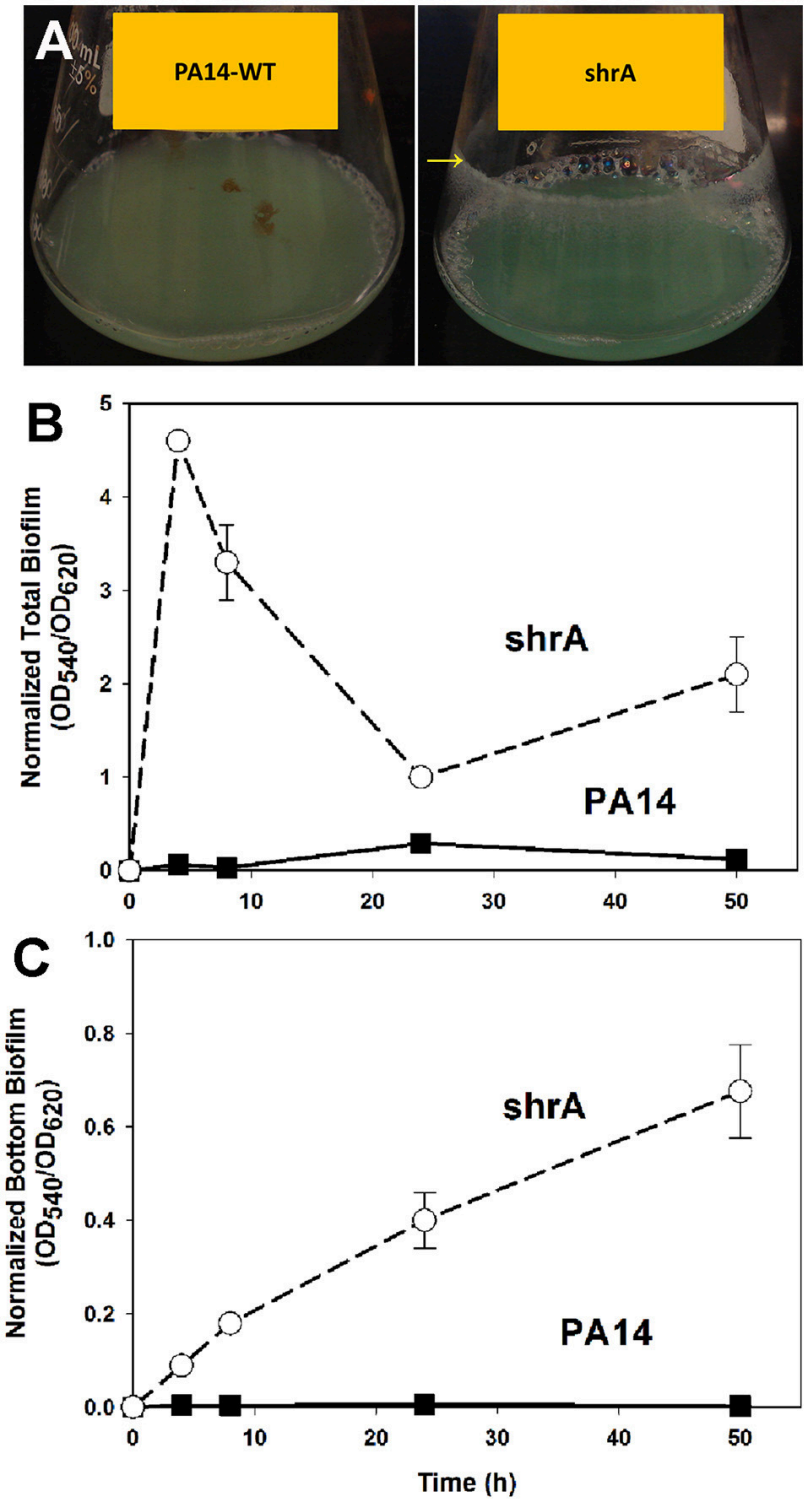
Inactivation of *shrA* increases biofilm formation in shake flasks and in microtitre plates. **(A)** The *shrA* mutant formed biofilms at the glass surface as indicated by the yellow arrow after overnight incubation in LB at 37°C with shaking at 250 rpm. Total biofilm formation (at the liquid/solid and air-liquid interfaces) **(B)**, and bottom biofilm formation on the polystyrene plates **(C)** by PA14 and the *shrA* mutant after incubation in LB at 37°C without shaking. Six wells were used for each culture. Error bars indicate standard deviations from three independent cultures.

The RSCV phenotype of the *shrA* mutant was associated with dramatically increased biofilm formation (Figure 3). In shake flasks, the *shrA* mutant developed biofilms at the air/liquid interface (Figure 3A) starting from an early growth stage (turbidity at 600 nm of 0.2). In polystyrene 96-well plates, the *shrA* mutant increased biofilm formation at the air/liquid interface as well as at the liquid/solid interface (Figures 3B,**C**). The increase was 76 ± 2-fold compared to PA14-WT after 4 h of incubation at 37°C and 119 ± 13-fold after 8 h incubation. This increase in biofilm formation occurred even though the *shrA* mutant grows at nearly the same rate (1.10 ± 0.08/h) as the wild-type (1.13 ± 0.00/h) in rich medium.

**Figure 4:**
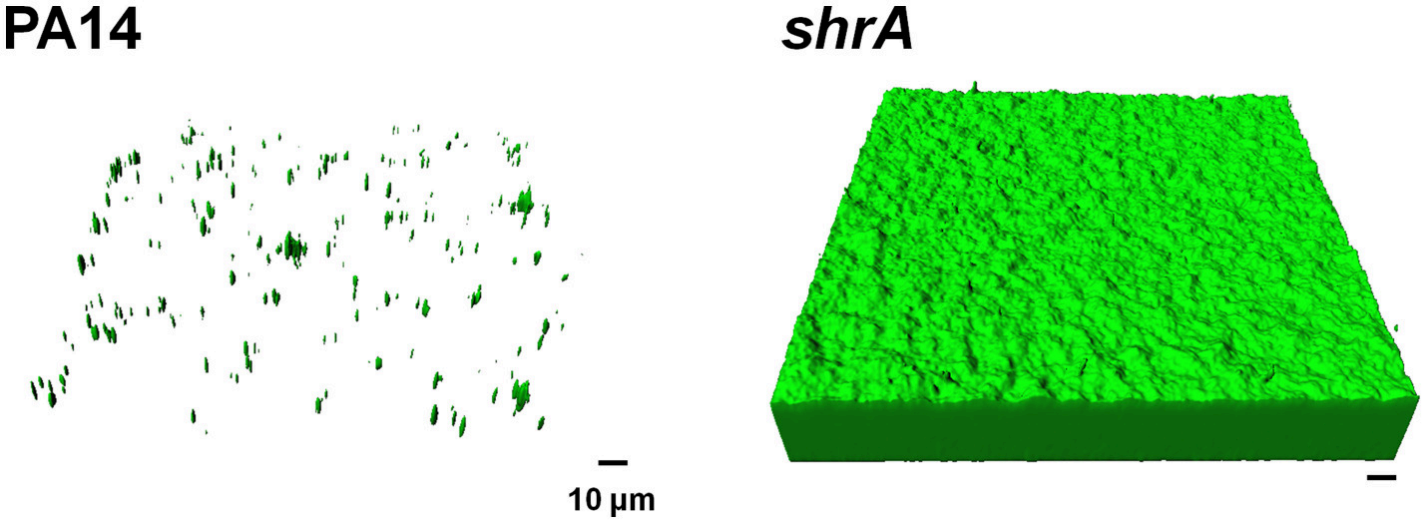
Inactivation of *shrA* increases biofilm formation in flow cells. Biofilms of *P. aeruginosa* PA14 and the *shrA* mutant were formed in flow cell chambers in 5% LB at 37°C. After 72 h of incubation, biofilms were stained with SYTO9 for 20 min in the dark. Random biofilm images were obtained using a confocal microscope, and the representative images shown were produced by IMARIS. Scale bar indicates 10 μm.

To corroborate the 96-well plate results, biofilm formation was tested using a flow cell assay with continuous flow of fresh medium (Figure 4). The biofilms formed by the *shrA* mutant have a 17-fold increase in biomass (2 ± 2 μm^3^/μm^2^ for PA14 vs. 33 ± 8 μm^3^/μm^2^ for the *shrA* mutant), a 7-fold increase in substratum coverage (13 ± 13% for PA14 vs. 96 ± 2% for the *shrA* mutant), and a 16-fold increase in thickness (2 ± 2 μm for PA14 vs. 32 ± 8 μm for the *shrA* mutant). In addition, the biofilm architecture of the *shrA* mutant was smoother and flatter than that of PA14 (roughness coefficient 1.6 ± 0.3 for PA14 vs. 0.06 ± 0.01 for the *shrA* mutant). Therefore, the *shrA* mutation dramatically increases biofilm formation.

### Inactivation of ShrA increases EPS while decreasing motility

**Figure 5:**
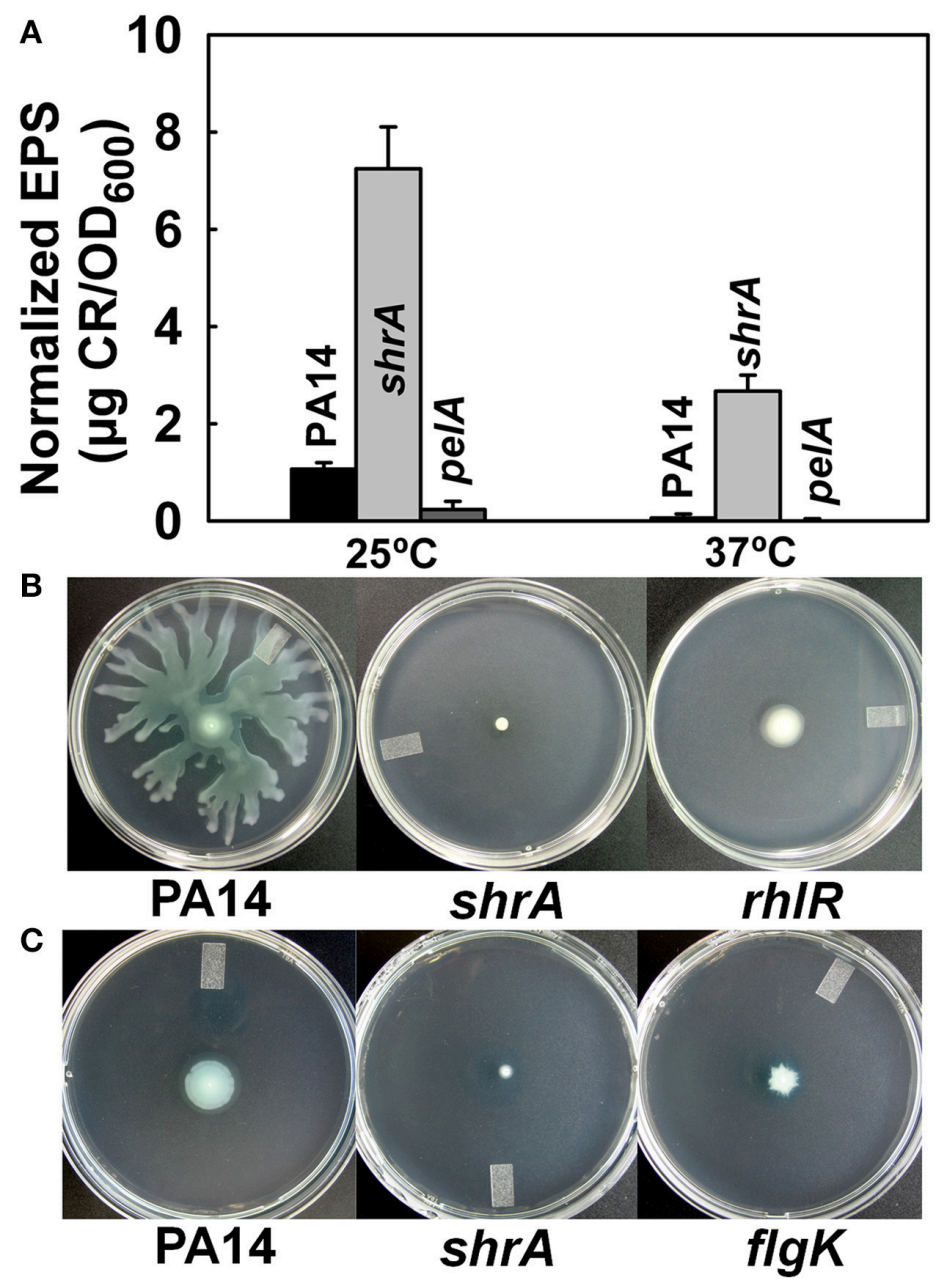
Inactivation of *shrA* increases EPS production and reduces motility. **(A)** EPS production by the Congo-red assay after incubation in LB for 24 h at 25°C or 16 h at 37°C. Error bars represent standard deviations from two independent cultures. **(B)** Swarming motility and **(C)** swimming motility of *P. aeruginosa* PA14 and the *shrA* mutant at 37°C after 24 h. Three plates were used for each culture and two independent cultures were used for each strain.

The red colony phenotype shown on the Congo-red plates of *shrA* mutant (Figure 2B) is due to Congo-red binding to EPS; hence, we quantified the amount of EPS bound to cells of PA14 and the *shrA* mutant at both 37°C and 25°C using a Congo-red binding assay and found the *shrA* mutant produces 45- and 7-fold more EPS than wild-type PA14 at 37° and 25°C, respectively (Figure 5A). The *pelA* mutant (negative control) did not form EPS at both temperatures tested. Considering that the Congo-red may not be an EPS-specific stain, we also used an anthrone-H_2_SO_4_ assay to test the EPS production. Corroborating the Congo-red assay results, the *shrA* mutation increased EPS production by 6.2 ± 0.4 compared to the wild-type strains at 37°C.

Reduced motility has been reported for other RSCVs (Ueda and Wood, 2009); hence, we examined motility for the *shrA* mutant; the *rhlR* (Köhler et al., 2000) and *flgK* (O'Toole and Kolter, 1998) mutants were used as negative controls for swarming and swimming motility, respectively. Although PA14 swarmed on the surface of plates at 24 h, the *shrA* mutation abolished swarming, like the *rhlR* mutation (Figure 5B). In addition, swimming motility was also reduced for the *shrA* mutant compared to the PA14 wild-type strain (Figure 5C). Therefore, the *shrA* mutation dramatically increases EPS, abolishes swarming, and decreases swimming.

### Inactivation of ShrA increases cellular c-di-GMP concentrations

**Figure 6:**
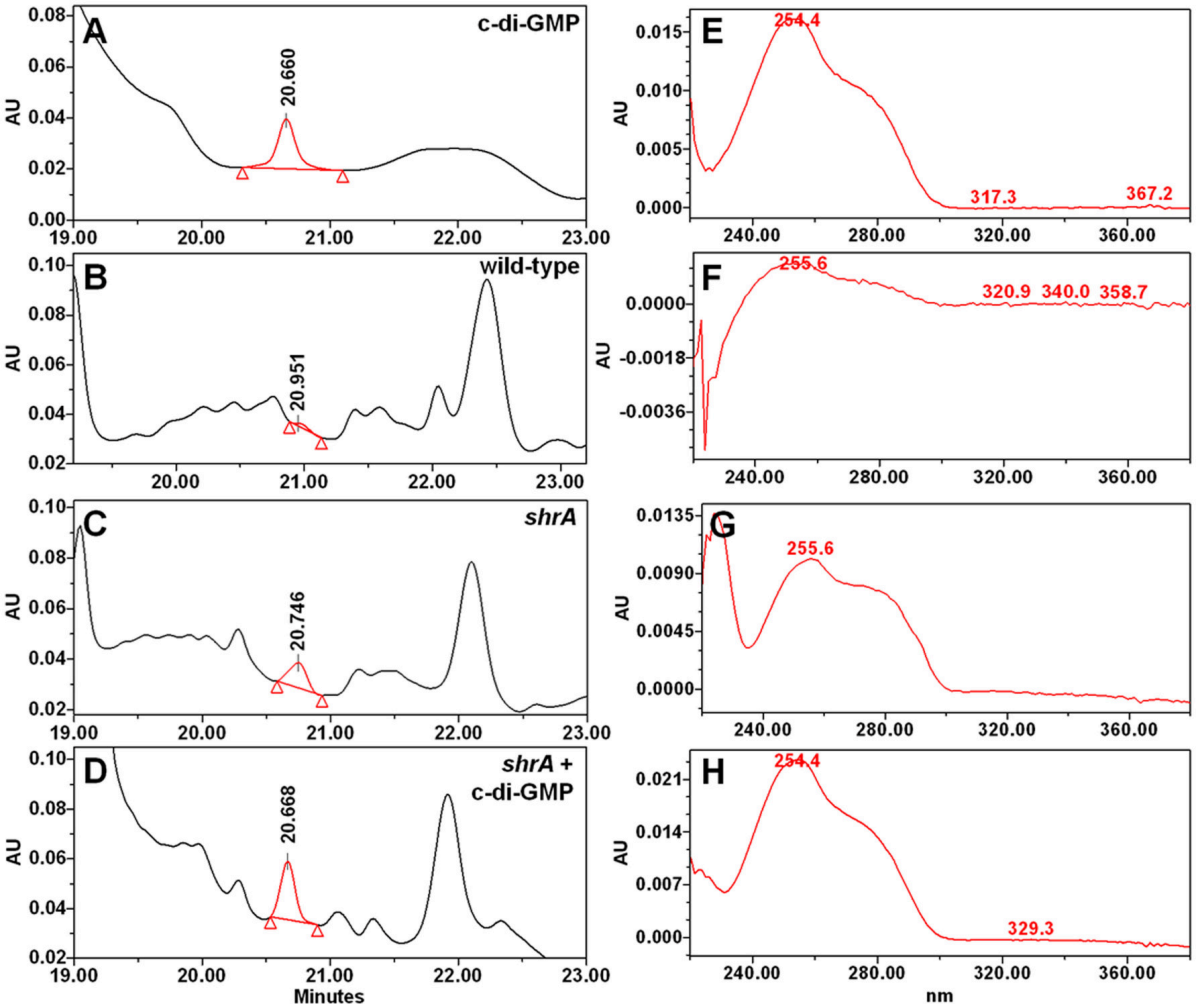
Inactivation of *shrA* increases c-di-GMP concentrations. Chromatography traces of **(A)** 10 μM synthetic c-di-GMP and nucleotide extracts from **(B)** *P. aeruginosa* PA14, **(C)** the *shrA* mutant, and **(D)** the *shrA* mutant spiked with 7.5 μM c-di-GMP. **(E–H)** spectra of the corresponding c-di-GMP peak of **(A–D)**, respectively.

RSCV morphology and the related phenotypes of increased biofilm, increased EPS, and reduced motility have been associated with increased concentrations of the second messenger c-di-GMP (Ueda and Wood, 2009); therefore, we measured the cellular c-di-GMP concentrations of PA14 and *shrA*. The *shrA* mutation increased c-di-GMP concentration 18 ± 3-fold (Figure 6). The cellular c-di-GMP level of the *shrA* mutant was 1.6 ± 0.2 pmol/mg cells. This is comparable to the c-di-GMP production of a small colony variant (around 2 pmol/mg cells) (Meissner et al., 2007). The c-di-GMP concentration of the wild-type strain was 0.09 ± 0.03 pmol/mg cells. This value is lower than the reported value (around 0.6 pmol/mg) for *P. aeruginosa* wild-type 20265 (Simm et al., 2009). However, the difference could be due to the strain difference as well as differences in growth conditions (we harvested the cells at a turbidity of 2.0 while the strain used for comparison was incubated in Luria-Bertani (LB) medium for 48 h). Therefore, the increased RSCV morphology, EPS, biofilm, and motility phenotypes of the *shrA* mutant may be explained by the increased c-di-GMP concentrations.

### Complementation of biofilm formation with ShrA

**Figure 7:**
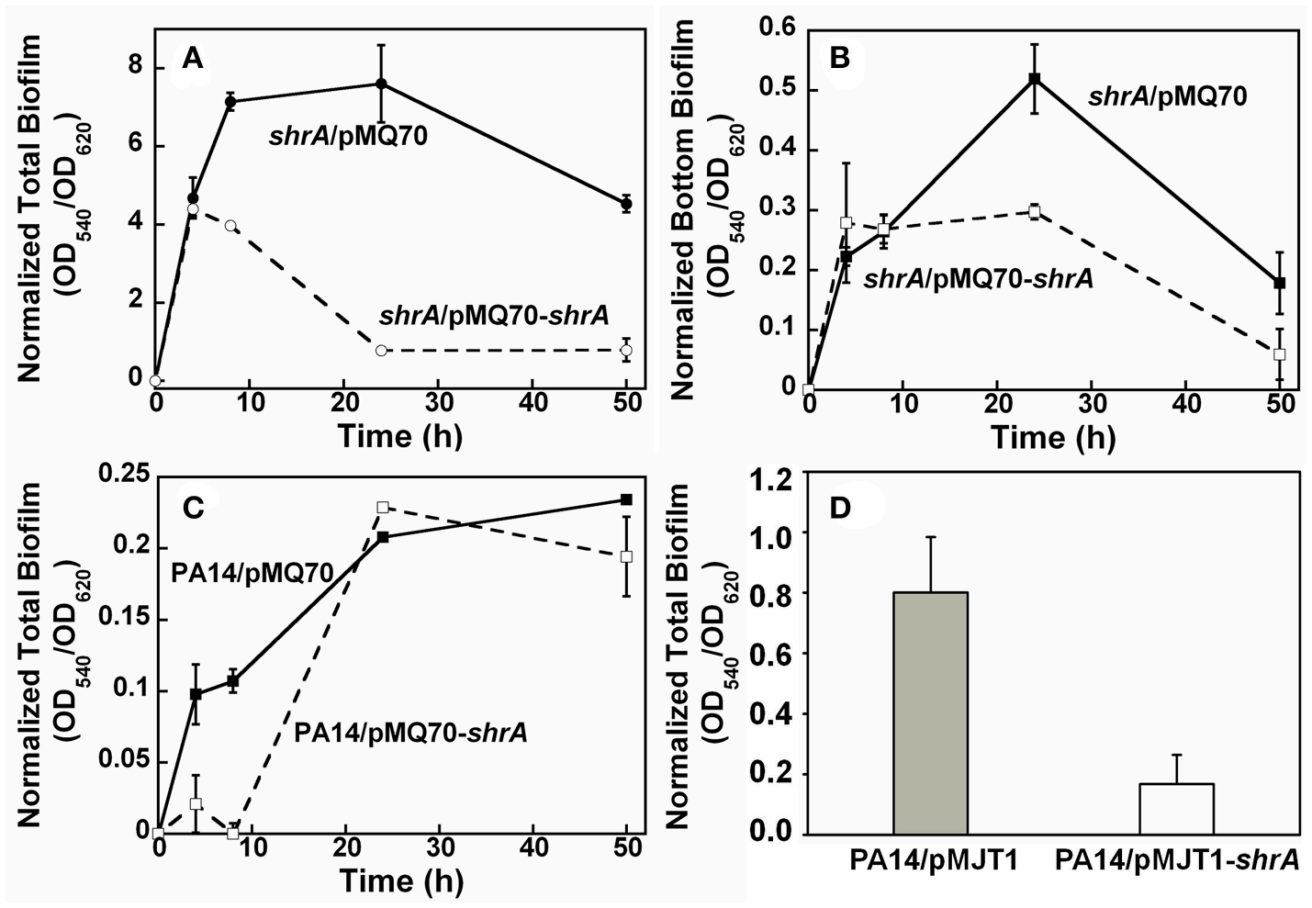
ShrA reduces biofilm formation. **(A)** Total biofilm formation and **(B)** bottom biofilm formation on polystyrene plates for production of ShrA in the *shrA* mutant via pMQ70-shrA, and **(C)** total biofilm formation on polystyrene plates for production of ShrA in *P. aeruginosa* PA14 via pMQ70-shrA. LB containing 0.05% arabinose at 37°C was used. **(D)** Total biofilm formation of *P. aeruginosa* PA14 on polystyrene plates for production of ShrA via pMJT1-shrA in LB containing 0.2% arabinose at 37°C for 24 h. Six wells were used for each culture and three independent cultures were used for each strain. Error bars represent the standard deviations.

To verify whether the phenotypes observed in the *shrA* mutant were caused by loss of function of ShrA, we confirmed the transposon insertion in *shrA* by PCR. Furthermore, complementation of the biofilm phenotype of the *shrA* mutation by producing ShrA production from a plasmid was investigated. As expected, *shrA* gene expression using plasmid pMQ70-*shrA* reduced total biofilm formation of the *shrA* mutant by 90% at 24 h (Figure 7A) and reduced the bottom biofilm formation by 43% (Figure 7B); *P. aeruginosa* forms biofilms along the sides of the 96-well plates as well on the bottom. Total biofilm formation of the PA14 strain was also delayed by *shrA* gene overexpression (Figure 7C). This result was confirmed by using another expression plasmid, pMJT1-*shrA*, from which expression of *shrA* reduced biofilm formation of PA14 strain 4.8 ± 2.9-fold after incubation in LB medium at 37°C for 24 h (Figure 7D). Therefore, the increased biofilm phenotype of the *shrA* mutation could be complemented.

### Genetic suppressor screening

To investigate how ShrA regulates the RSCV phenotype, genetic screening was conducted using Tn*5-luxAB* transposon mutagenesis to find suppressive loci for the RSCV phenotype from the *shrA* mutation. The double mutant library (*shrA* plus random gene inactivation) was first screened for a reduction in aggregation and only the cells remaining in the supernatant that failed to aggregate like the *shrA* mutant were grown on Congo-red plates with selective antibiotics; approximately 5,000 double mutant colonies were screened. After incubating at 37°C for 2 days, colonies displaying a white and smooth shape like the wild-type strain were chosen. Eleven of these colonies were sequenced from the transposon: four of these had the Tn5-*luxAB* insertion in the *pel* locus (Table 1), and one had the insertion in the PA0839 gene. Critically, there were six suppressive mutations in four genes of the *wsp* chemosensory system: *wspA, wspD, wspE*, and *wspR* (Table 1). Therefore, the *shrA* mutation appears to cause the RSCV phenotype through an alteration of the Wsp chemosensory system.

**Table 1:** Suppressive loci for the *shrA* mutation.

**Strain**	**PAO1 ID**	**PA14 ID**	**Gene name**	**Gene function**
Suppressor 23	PA3702	PA14_16500	*wspR*	WspR, diguanylate cyclase/response regulator
Suppressor 8	PA3704	PA14_16470	*wspE*	WspE, histidine kinase/response regulator
Suppressor 9	PA3704	PA14_16470	*wspE*	
Suppressor 6	PA3705	PA14_16460	*wspD*	WspD, scaffold protein, regulates WspE activity
Suppressor 10	PA3705	PA14_16460	*wspD*	
Suppressor 29	PA3708	PA14_16430	*wspA*	WspA, a membrane bound methyl-accepting chemotaxis protein, will activate WspE when methylated.
Suppressor 3	PA3058	PA14_24560	*pelG*	Predicted membrane protein related to EPS production, PelG
Suppressor 24	PA3061	PA14_24510	*pelD*	*L*-lactate permease related to EPS production, PelD
Suppressor 4	PA3064	PA14_24480	*pelA*	Oligogalacturonide lyase related to EPS production, PelA
Suppressor 25	PA3064	PA14_24480	*pelA*	Oligogalacturonide lyase related to EPS production, PelA
Suppressor 11	PA0839	PA14_53410		Probable transcriptional regulator

*Genetic suppressor screening identified additional mutations that mask the phenotypes of the shrA mutant*.

### ShrA is an oligomer containing one PLP per dimer

The physiological role of SHMT is to catalyze the reversible conversion of glycine and (6*S*)-5,10-CH_2_-THF to l-serine and (6*S*)-THF (Schirch and Szebenyi, 2005; Figure 1A). (6*S*)-5,10-CH_2_-THF provides the largest part of the one-carbon units available to the cell (Stover and Schirch, 1990) and is a precursor for *S*-adenosylmethionine (SAM) synthesis through the folate pathway (Figure 1A; Shoeman et al., 1985). Since the methyltransferase WspC (analog to CheR) uses SAM to methylate the receptor WspA (Springer and Koshland, 1977), we hypothesized that inactivation of ShrA may increase the availability of the one-carbon units which will drive the methylation of WspA.

To check the hypothesis, we characterized the *in vitro* activity of the purified enzyme. Recombinant ShrA with a 6X-His tag at the carboxy terminus was produced in *E. coli* BL21 (DE3), and the protein was purified (>95%) (Figure S1A). For SHMT, the dimer is the minimum necessary structure for the catalytic activity (Bhatt, Bhakuni, Kumar, Khan and Siddiqi); SHMT from *E. coli* as well as that from several bacterial sources is dimeric whereas SHMT from mammalian sources is a homotetramer. We checked the oligomerization state of ShrA by glutaraldehyde cross-linking and found ShrA shifted from a monomer to an oligomer after cross-linking using glutaraldehyde as determined by SDS-PAGE (Figure S1B). The oligomer protein band was not a sharp band of dimer at 90 kDa but was broad, indicating possible crosslinking products from a dimer to a tetramer, or an oligomer with intra-molecular cross-linking by glutaraldehyde. Therefore, ShrA from *P. aeruginosa* PA14 is present as an oligomer as are other bacterial SHMTs.

SHMT is a PLP-dependent enzyme in which PLP is covalently attached to the enzyme (Chaturvedi and Bhakuni, 2003). SHMT from *E. coli* as well as SHM2 from *M. tuberculosis* contain 2 PLP/(mol enzyme dimer) (Chaturvedi and Bhakuni, 2003); however, SHM1 from *M. tuberculosis* contains only 1 PLP/(mol enzyme dimer) (Chaturvedi and Bhakuni, 2003). Therefore, we checked the PLP content for ShrA. The molar ratio of PLP to ShrA monomer is 0.5 ± 0.1 as calculated from four samples. This ratio is similar to that of SHM1 from *M. tuberculosis*, but different from other bacterial SHMTs.

### ShrA primarily produces ser and (6*S*)-THF from Gly and (6*S*)-5,10-CH_2_-THF

**Figure 8:**
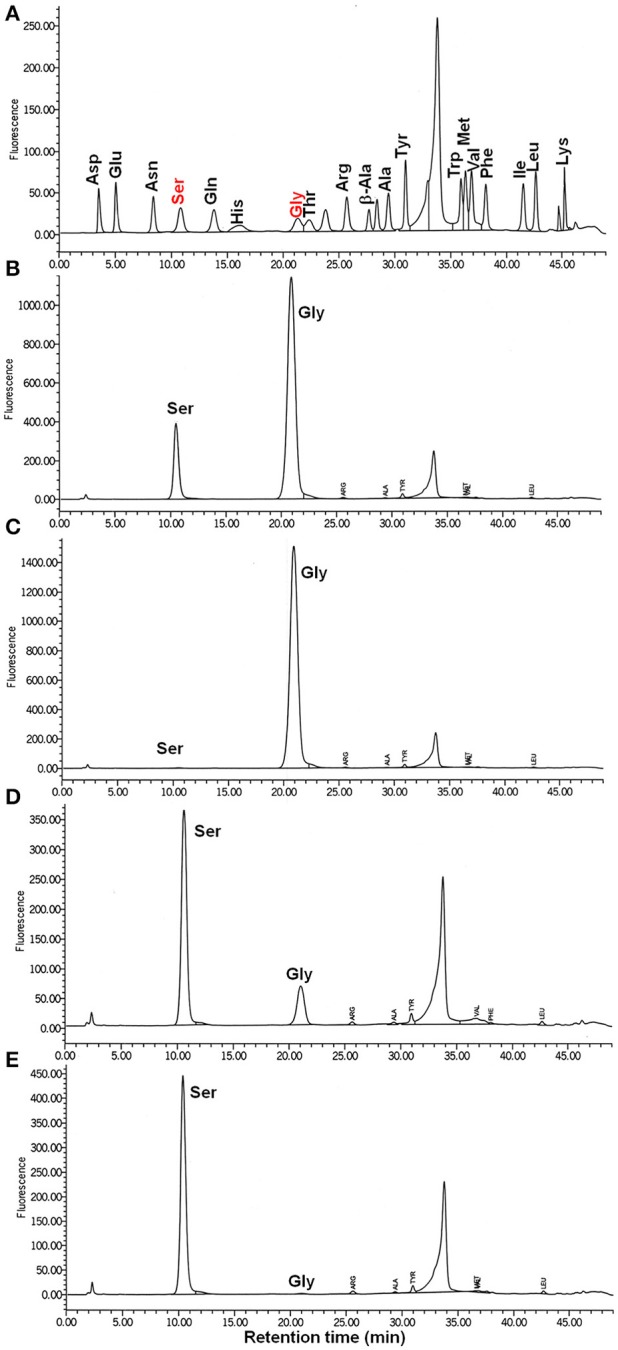
ShrA produces primarily Ser not Gly via the HPLC-fluorometric assay. **(A)** HPLC standards for 10 μM of each amino acid. **(B)** Ser is the product of the forward reaction of Gly (20 to 30 mM) + (6*R,S*)-5,10-methylenetetrahydrofolate (2 to 3 mM). **(C)** Negative control (buffer, no ShrA) for the forward reaction: Gly + (6*R,S*)-5,10-methylenetetrahydrofolate was not converted to Ser. **(D)** Gly is the product of the backward reaction of Ser (4–6 mM) and (6*R,S*)-tetrahydrofolate (2–3 mM): Ser was converted to Gly. **(E)** Negative control (buffer, no ShrA) for the backward reaction: Ser + (6*R,S*)-tetrahydrofolate was not converted to Gly.

According to our hypothesis, ShrA should primarily catalyze the conversion of Gly and (6*S*)-5,10-CH_2_-THF to Ser and (6*S*)-THF instead of the reverse reaction (Figure 1A) for the production of one-carbon units as its pivotal role (Rao et al., 2003; Florio et al., 2010). To confirm this, we characterized the enzyme activity *in vitro* toward both Ser/(6*S*)-THF and Gly/(6*S*)-5,10-CH_2_-THF using purified ShrA*;* Figure 8A shows the chromatogram of each amino acid separated by high-performance liquid chromatography (HPLC). For the forward reaction (Figure 1A), the substrates Gly (20 mM) and (6*R,S*)-5,10-CH_2_-THF (2 mM) were reacted with ShrA, and the product (6*S*)-THF was immediately recycled back to the substrate (6*S*)-5,10-CH_2_-THF by using excess formaldehyde, so the substrate (6*R,S*)-5,10-CH_2_-THF concentration was constant throughout the reaction. The product Ser was quantified (Figure 8B), and protein dialysis buffer instead of the protein was used as the negative control (Figure 8C). The enzyme specific activity was calculated to be 4.24 ± 0.01 μmol/min/mg. In order to confirm that the value represents V_max_, we also increased the substrate concentrations to 30 mM Gly and 3 mM (6*R,S*)-5,10-CH_2_-THF, and the activity was determined to be similar (4.01 μmol/min/mg).

For the reverse reaction, the amount of Gly produced from the substrates Ser (4 mM) and (6*R,S*)-THF (2 mM) was analyzed (Figure 8D), and the specific activity was 1.6 ± 0.3 μmol/min/mg. Protein dialysis buffer instead of the protein was used as the negative control (Figure 8E). When the concentrations of the substrates were increased to 6 mM Ser and 3 mM (6*R,S*)-THF, the specific activity was nearly identical (1.5 ± 0.3 μmol/min/mg); hence, these values are equivalent to V_max_. Therefore, ShrA is active as an SHMT, and the V_max_ is 2.8-fold higher for converting Gly/(6*S*)-5,10-CH_2_-THF to Ser/(6*S*)-THF than for the reverse direction.

### The *shrA* operon also encodes proteins to decrease Gly and produce (6*S*)-5,10-CH_2_-THF

The *shrA* operon encodes genes for both Ser and Gly metabolism (Figure 1B), including those for the glycine cleavage system (GcvH2/P2/T2) and a serine dehydratase (SdaA). The GCV system is responsible for the degradation of glycine and converts (6*S*)-THF to (6*S*)-5,10-CH_2_-THF (Figure 1A), so the GCV system is an additional source for the one-carbon units. Therefore, the proteins encoded by this operon should regulate the two amino-acid metabolism cooperatively. To investigate this, we checked the colony morphology of the transposon mutants (Figure 2C) and found that all of these mutants have the same morphology as the wild-type strains. Therefore, only ShrA inactivation leads to RSCV in the *shrA* operon since only this mutation should increase the one-carbon source (6*S*)-5,10-CH_2_-THF.

### Differentially regulated genes in biofilm cells of the *shrA* mutant

To explore the mechanism for ShrA regulation and the impact of ShrA inactivation on the whole genome (in addition to the Wsp chemosensory system), a whole-transcriptome analysis was performed with biofilm cells on glass wool for the *shrA* mutant after incubating at 37°C for 7 h. There were 24 genes found to be induced for the *shrA* mutant biofilm more than 1.8-fold, 12 of which were related to phage. In contrast, 172 genes were found to be repressed in *shrA* biofilm cells more than 1.8-fold. Among them, 21 genes were related to denitrification (Table S1), such as the gene that encodes nitrite reductase *nirS*, genes encode nitric oxide reductase *norBCD*, and the gene encodes nitrous reductase *nosZ*. Another large group of affected genes is related to iron acquisition: there are total 65 genes in this group and 41 of these were related to siderophores, including the *pvd* gene clusters encoding for pyoverdine production, and the *pch* gene cluster encoding pyochelin. Another 13 genes were related to redox enzymes; for example, some of the FMNH_2_/NAD dependent proteins such as SsuDE and some oxygenases were repressed. The common feature for the encoded proteins is that they are related to electron transport and might be regulated by cell redox homeostasis.

### ShrA decreases the NADH/NAD^+^ ratio

Based on the transcriptome results, we examined the role of ShrA on redox homeostasis by investigating the NADH/NAD^+^ ratio since it is representative of the intracellular redox state (Price-Whelan et al., 2007). We utilized bacteria grown on agar surfaces since colonies have been used to study the development of cell communities (Ramos et al., 2010) and found that the *shrA* mutation increased the NADH/NAD^+^ ratio from 0.25 ± 0.04 for the wild-type strain to 0.50 ± 0.03. Therefore, the intracellular NADH/NAD^+^ ratio was increased 2.0 ± 0.3-fold by the *shrA* mutation.

### Nitrate abolished the *shrA* rugose phenotype as an electron acceptor

To check whether the enhanced NADH/NAD^+^ ratio plays a role in the rugose phenotype of the *shrA* mutant, we examined the effect of nitrate (${\text{NO}}_{3}^{-}$) on *shrA* colony morphology. ${\text{NO}}_{3}^{-}$ is a terminal electron acceptor (Williams et al., 1978), and it reduces cellular NADH/NAD^+^ levels (Price-Whelan et al., 2007) while it does not affect the total concentrations of NADH and NAD^+^ (Price-Whelan et al., 2007; Figure 1A). Addition of 30 mM KNO_3_ (a physiologically-relevant concentration, Price-Whelan et al., 2007) completely converted the *shrA* rugose colony into the smooth colony morphology of the wild-type strain (Figure 2B). In comparison, the rugose phenotype of another RSCV mutant, *tpbA* (Ueda and Wood, 2009), was only partially reduced by nitrate (Figure 2B). Therefore, the increased NADH/NAD^+^ ratio caused by inactivating ShrA positively regulates the RSCV phenotype.

### ShrA increases pyocyanin levels

To further investigate the role of ShrA in redox homeostasis, we investigated its effect on pyocyanin levels; pyocyanin is secreted and generates reactive oxygen species (Bodelón et al., 2016). We found that inactivating ShrA led to a 2.4 ± 0.4-fold increase in pyocyanin levels. As expected, the negative control *phzS* reduced pyocyanin 5.1 ± 3.3-fold In agreement with our results, increased levels of c-di-GMP have been shown to increase pyocyanin levels (Lo et al., 2016). Note that reduced transcription of *shrA* via inactivation of PA2449 (GcsR), a positive regulator of the operon that includes *shrA*, has also been reported to reduce pyocyanin (Lundgren et al., 2013), but this was later shown to likely be due a *P. aeruginosa* PAO1 strain artifact (Sarwar et al., 2016).

## Discussion

### Inactivation of the SHMT ShrA leads to increased c-di-GMP

In this study, we demonstrated that ShrA is an SHMT that regulates the RSCV phenotype of *P. aeruginosa;* this is the first biochemical characterization of this enzyme in *P. aeruginosa*. The RSCV phenotype for the *shrA* mutant and the related hyper-biofilm formation, increased EPS production, and reduced swarming motility may be explained by the 20-fold increased intracellular c-di-GMP concentrations, that we measured (Figure 6). c-di-GMP is an ubiquitous intracellular second messenger that acts as a central regulator in bacterial physiology, especially in regulating the transition between motile and sessile states (Hengge, 2009), and there is ample evidence to correlate RSCV with increased c-di-GMP concentrations (Hickman et al., 2005; Ueda and Wood, 2009). For example, inactivation of protein tyrosine phosphatase TpbA caused RSCV formation through constitutive activation of a DGC TpbB, which results in elevated c-di-GMP concentrations (Ueda and Wood, 2009; Pu and Wood, 2010).

### ShrA controls c-di-GMP through the Wsp chemosensory system

To examine how the SHMT ShrA controls c-di-GMP concentration, we screened rugose suppressor mutants (Table 1) and identified the *wsp* two-component chemosensory system (*wspADER*) is a necessary component downstream of ShrA for causing the RSCV phenotype. WspR is a DGC containing a CheY domain and a GGDEF domain, which catalyzes the production of c-di-GMP (Hickman et al., 2005). Deletion of *wspF* in *P. aeruginosa* causes the constitutive activation of WspR that leads to the rugose phenotype (Hickman et al., 2005). By comparing the Wsp system to the well-studied Che chemotaxis system, the signal cascade was predicted (Figure 1C; Bantinaki et al., 2007). Upon an external signal, the membrane bound methyl-accepting chemotaxis protein (MCP), WspA, is methylated which activates autophosphorylation of the histidine kinase WspE. The methylation of WspA is controlled by the opposing activities of the methyltransferase WspC and the methylesterase WspF. The phosphorylated WspE relays the phosphate group to the CheY domain of WspR which activates its DGC activity. The predicted functions of WspC and WspF have been confirmed in *P. fluorescens* (Bantinaki et al., 2007) in that deletion of *wspF* or overexpression of *wspC* will convert a smooth colony into a rugose colony.

The suppressor mutants we identified in *wspA, wspD, wspE*, and *wspR* are consistent for the signal cascade (Figure 1C) in that WspA, WspD and WspE are all positive regulators of WspR as a DGC. Of significance is that it indicates ShrA controls cellular c-di-GMP concentrations through the DGC WspR, especially by influencing WspA, the most upstream component among these four genes. PA14 has 37 putative c-di-GMP related proteins, including 16 proteins with a GGDEF domain, 5 with a PDE domain, and 16 that contain both domains; however, not all of these proteins with DGC and PDE domains are active enzymes since some of the domains are used for regulation (Kulasakara et al., 2006). Hence, among the GGDEF proteins, only PA1107, TpbB, WspR, and PA5487 increased biofilm formation when overexpressed (Kulasakara et al., 2006). Previously, using the same phenotype suppressor screening for the *tpbA* RSCV mutant, only the DGC TpbB was found downstream (Ueda and Wood, 2009). In contrast, for this *shrA* mutant, only the WspR system was identified downstream rather than the other DGCs. Hence, ShrA controls c-di-GMP concentrations through the Wsp system rather than other DGCs. Perhaps these two means of controlling c-di-GMP for biofilm formation (Wsp and TpbB) are distinct and arise based on the use of two different appendages for surface sensing.

### ShrA reduces one carbon equivalents to reduce rugose cells

The common concept for the physiological function of SHMT is that this enzyme is utilized by the cell for generating one-carbon fragments to (6*S*)-5,10-CH_2_-THF for the synthesis of diverse metabolites (Rao et al., 2003; Florio et al., 2010). However, the *in vitro* ShrA enzyme activity indicates that ShrA prefers converting Gly/(6*S*)-5,10-CH_2_-THF to Ser/(6*S*)-THF rather than the reverse reaction. Therefore, ShrA reduces the availability of one-carbon units.

According to this physiological function of ShrA, we propose the mechanism of how it controls the RSCV phenotype shown in Figure 1A. Inactivation of ShrA leads to accumulated (6*S*)-5,10-CH_2_-THF, which provides the one-carbon building blocks. Delivering the one-carbon source to SAM via the folate pathway (Shoeman et al., 1985) (Figure 1A), including 5,10-methylenetetrahydrofolate reductase MetF, methionine synthase MetH, and methionine methyltransferase MetK, allows SAM to transfer the methyl group to WspA by the methyltransferase WspC, which activates the DGC WspR.

The non-rugose phenotype of the *gcv* mutants (Figure 2C) also supports our hypothesis. The GCV system produces (6*S*)-5,10-CH_2_-THF (Figure 1B). Thus, inactivation of the GCV system should lead to the reduction of the one-carbon units rather than their accumulation as the *shrA* mutation did. Encoding the GCV system together with ShrA in the same operon seems to keep the supply of active one-carbon units balanced while inactivation of *shrA* alters this balance. Of course, the impact of Gly on cell physiology is complex; for example, we found that 200 mM Gly inhibited the growth of PA14 in tryptone medium at 25°C, and high concentrations of glycine inhibit the growth of many bacteria (Ratomahenina et al., 1979; Minami et al., 2004). A possible mechanism for this inhibition is that UDP-*N*-acetylmuramate-alanine ligase activity is reduced by glycine and thus cell wall component synthesis is impaired (Minami et al., 2004). It is also interesting to note that a glycine resistant mutant of *Pseudomonas stutzeri* entails increased activity of the serine hydroxymethyltransferase (Ratomahenina and Galzy, 1981) which would reduce Gly concentrations which is consistent with our mechanism.

### Cell redox state plays a role in the RSCV phenotype via ShrA

The increase in the NADH/NAD^+^ ratio is consistent with our proposed mechanism (Figure 1A); inactivation of ShrA prevents the catabolism of Gly through the ShrA pathway which should enhance the GCV pathway and lead to increased NADH. The increased NADH levels then serve as a cofactor of MetF for the production of 5-methyltetrahydrofolate [(6*S*)-5-CH_3_-THF] and thus increase the availability of one-carbon units. The fact that addition of the electron acceptor ${\text{NO}}_{3}^{-}$ abolished the rugose morphology of the *shrA* mutant but not the *tpbA* mutant (Figure 2B) indicates that the altered NADH/NAD^+^ ratio plays a key role for the rugose phenotype of the *shrA* mutant and that NADH is a required factor rather than a side effect. Hence, inactivation of ShrA alters the cell redox state, which cooperatively regulates colony morphology through the folate pathway and the Wsp system.

The redox state of *P. aeruginosa* has been linked to colony morphology before since a *phz* mutation enhances the NADH/NAD^+^ ratio (Price-Whelan et al., 2007) and results in large rugose colonies at 20°C (Dietrich et al., 2008). However, the regulation mechanism has not been elucidated yet. Our finding links the redox state and a downstream diguanylate cyclase, WspR, which further regulates the rugose morphology through the ubiquitous second messenger c-di-GMP.

### Inactivation of ShrA represses iron acquisition

Our whole transcriptome analysis of the *shrA* biofilm cells linked this mutation to denitrification, iron acquisition, and the redox state (Table S1). However, for the *wspF* mutant, which also results in RSCV and increased c-di-GMP due to increased WspR activity, these same genes were differently regulated (Hickman et al., 2005). For example, the *shrA* mutation repressed genes for denitrification (*nir, nor* and *nos* gene clusters) while these genes were not changed by the *wspF* mutation. Hence, the repression of these genes in *shrA* mutant is not simply due to increased c-di-GMP levels; instead, they were regulated due to the redox change upstream of the Wsp system.

One common feature for the iron acquisition, denitrification and redox genes repressed in the transcriptome analysis is that they could all be directly or indirectly related to electron transport or the cell redox state. Indeed, an increased intracellular NADH/NAD^+^ ratio was observed for the *shrA* mutant, indicating a more reduced cellular environment. A large group of genes that were repressed upon inactivating ShrA were those related to iron acquisition, including siderophore-related genes (Table S1). In *P. aeruginosa*, the expression of these iron acquisition genes is strictly regulated in response to the environmental iron concentrations through the ferric uptake regulator (Fur) protein (Ochsner and Vasil, 1996). At high iron concentrations, Fur binds to Fe^2+^ and the Fur-Fe^2+^ complex represses these genes. For the *shrA* mutant, various Fur-regulated genes were found to be repressed along with the *pvd* and *pch* genes, including *pfeR, tonB, fumC, fpvA, phuR, fiuA, pirA, piuA*, and *fptA* (Ochsner and Vasil, 1996; Cornelis et al., 2009). Since the *shrA* cells are in a more reduced state with higher NADH levels and since NADH (redox potential ${\text{E}}_{0}^{\prime }$ = −320 mV) is capable of reducing Fe^3+^ to Fe^2+^ (${\text{E}}_{0}^{\prime }$ = 770 mV) (Berg et al., 2002), it is reasonable to postulate that the Fe^2+^/Fe^3+^ ratio increased upon inactivating ShrA so that there is more Fe^2+^ accessible for Fur, which leads to the repression of these iron acquisition genes. Hence, the altered redox state may be the cause the repression of the iron acquisition genes.

### Inactivation of ShrA represses denitrification

The transcriptome analysis with the *shrA* mutant also revealed the denitrification gene clusters (*nir, nor*, and *nos* genes) were repressed. The expression of the denitrification machinery of *P. aeruginosa* is affected by redox signaling through the transcription factors ANR (anaerobic regulation of arginine deiminase and nitrate reduction) and DNR (dissimilative nitrate respiration regulator) (Giardina et al., 2008). In *anr* or *dnr* mutants, *nirS* and *norCB* transcription is repressed (Arai et al., 1995). ANR is regulated by the redox state through the [4Fe-4S]^2+^ cluster oxygen sensor while DNR is an NO sensor through the cofactor ferrous heme (Giardina et al., 2008). The redox state of the cofactors [4Fe-4S]^2+^ and ferrous heme may be altered by the increased NADH levels in the *shrA* mutant. Hence, the altered redox state may explain the repression of the denitrification genes.

### ShrA regulation

SHMT could be regarded as the initial enzyme of a branched pathway, and is likely to involve complex regulation (Dev and Harvey, 1984b). Using *E. coli* auxotrophic cultures, Harvey et al. found that SHMT synthesis is subject to active control mechanisms which respond to the requirements for various end products of the folate pathway, for purine biosynthesis, and for methylation reactions, as well as to serine limitation (Dev and Harvey, 1984b). In *E. coli*, the rate of SHMT synthesis is a hyperbolic function of (homo-Cys/SAM) (Dev and Harvey, 1984a), thus it is postulated that homo-Cys acts as an inducer of SHMT and SAM acts as a corepressor, suggesting that it is the requirements of methionine for methylation reactions which controls the SHMT synthesis (Dev and Harvey, 1984a).

The regulation of the SHMT ShrA in *P. aeruginosa* is not clear yet, but it is also likely to be complicated. *shrA* was identified to be a QS induced gene in PAO1 by Schuster et al. (Schuster et al., 2003) by comparing the transcriptome of the wild-type strain with a *lasRI rhlR* mutant whose QS was repressed. The *gcv* genes in the same operon were also found to be induced by QS. *shrA* is also repressed 955-fold in *ppyR* (PA2663) biofilm cells compared to the PAO1 wild-type (Attila et al., 2008). Since the *ppyR* mutant also repressed QS genes, the repression of *shrA* could be further linked to QS. For the microarray data of the *shrA* mutant biofilm cells, the *shrA* gene as well as two other genes in the same operon, *gcvT2* and *sdaA*, were all repressed comparing with the PA14 wild-type. Hence, the *shrA* gene in *P. aeruginosa* should be highly regulated.

Overall, we have discovered insights into how central metabolism; i.e., the conversion of the amino acids glycine to serine by hydroxymethyltransferase ShrA, impact the sessile lifestyle and virulence of the opportunistic pathogen *P. aeruginosa*. Furthermore, we demonstrate that the mechanism by which ShrA impacts biofilm formation is via reduction of c-di-GMP through its reduction of the intermediates glycine, (6*S*)-5,10-CH_2_-THF, methionine, and *S*-adenosylmethionine (Figure 1A).

## Materials and methods

### Strains and growth conditions

All strains and plasmids used in this study are listed in Table 2. *P. aeruginosa* PA14 (wild-type) and its isogenic mutants were obtained from the Harvard Medical School (Liberati et al., 2006). *P. aeruginosa* and *Escherichia coli* were grown in LB medium (Sambrooke et al., 1989) at 37°C unless noted. Gentamicin (15 μg/mL) was used for growth of the *P. aeruginosa* transposon mutants, carbenicillin (300 μg/mL) was used to maintain *P. aeruginosa* plasmid pMQ70 and pMJT1, and kanamycin (50 μg/mL) was used to maintain *E. coli* plasmid pET28b. Transposon insertion of the *shrA* mutant was verified as described previously (Ueda and Wood, 2009). Briefly, the PCR product amplified using primers PA14_33010-VF and PA14_33010-VR (Table 3) from the chromosomal DNA of *shrA* mutant was 1 kb larger than that from the wild-type, which corresponds to the size of the transposon. In addition, the DNA fragment corresponding to the end of the transposon and *shrA* gene was amplified with *shrA* chromosomal DNA using primers PA14_33010-VF and GB-3a (Table 3) and PA14_33010-VR and R1 (Table 3) but these pairs of primers did not amplify PA14 wild-type chromosomal DNA.

**Table 2:** Strains used in this study.

**Strain**	**Genotype or description**	**Reference**
***P. aeruginosa***		
PA14	Wild-type strain	Liberati et al., 2006
PA14_33010 (PA2444, *shrA*)	PA14_33010 Ω *Mar2xT7*, Gm^R^	Liberati et al., 2006
PA14_19120 (PA3477, *rhlR*)	PA14_19120 Ω *Mar2xT7*, Gm^R^	Liberati et al., 2006
PA14_50360 (PA1086, *flgK*)	PA14_50360 Ω *Mar2xT7*, Gm^R^	Liberati et al., 2006
PA14_24480 (PA3064, *pelA*)	PA14_24480 Ω *Mar2xT7*, Gm^R^	Liberati et al., 2006
PA14_13660 (PA3885, *tpbA*)	PA14_13660 Ω *Mar2xT7*, Gm^R^	Liberati et al., 2006
PA14_33040 (PA2442, *gcvT2*)	PA14_33040 Ω *Mar2xT7*, Gm^R^	Liberati et al., 2006
PA14_33030 (PA2443, *sdaA*)	PA14_33030 Ω *Mar2xT7*, Gm^R^	Liberati et al., 2006
PA14_33000 (PA2445, *gcvP2*)	PA14_33000 Ω *Mar2xT7*, Gm^R^	Liberati et al., 2006
***E. coli***		
BL21(DE3)	F^−^*ompT hsdS_*B*_(${\text{r}}_{B}^{-}$${\text{m}}_{B}^{-}$) gal dcm λ*(DE3) Ω p*lac*UV5*::*T7 polymerase	Novagen
HB101	*pro leu thi lacY* Str*^*r*^ endoI^−^ recA^−^ r^−^ m^−^*	Ditta et al., 1980
S17-1(*λpir*)/pUT-miniTn*5*-*luxAB*	Tc^R^ Sm^R^ Tp^R^ *mod*^+^ *res thi pro recA hsdR17* Ω RP4-TC::Mu-Km::Tn7 with pUT-miniTn*5-luxAB*	Simon et al., 1983; Ramsey and Whiteley, 2004
TG1	K12, *lac–pro supE thi hsdD5* (F' *traD36 proA*^+^*B*^+^ *lacI*^q^ *lacZ* M15)	Sambrooke et al., 1989
**PLASMIDS**		
pMQ70	Car^R^, Ap^R^, P_BAD_, expression vector	Shanks et al., 2006
pMQ70-*shrA*	Car^R^, Ap^R^, P_BAD_::*shrA*, complementation plasmid	This study
pMJT1	Car^R^, Ap^R^, P_BAD_, expression vector	Olsen et al., 1982
pMJT1-*shrA*	Car^R^, Ap^R^, P_BAD_::*shrA*, complementation plasmid	This study
pRK2013	Mobilizing conjugation plasmid	Figurski and Helinski, 1979
pET28b	Km^R^, P_T7_ expression vector	Novagen
pET28b-PA2444cHis	Km^R^, P_T7_::*shrA-cHis*^+^, expression vector for ShrA-cHis	This study

*Gm^R^, Tc^R^, Km^R^, Car^R^, Cm^R^, and Ap^R^ indicate gentamicin, tetracycline, kanamycin, carbenicillin, chloramphenicol, and ampicillin resistance, respectively*.

**Table 3:** Primers used in this study.

**Name**	**Sequence (5′-3′)**	**Description**
PA14_33010-VF	TGACACCGACACGACAAGAG	Verification of *shrA* tn insertion
PA14_33010-VR	GATAGCCCTCGGCATACTTG	Verification of *shrA* tn insertion
GB-3a	TACAGTTTACGAACCGAACAGGC	Verification of *shrA* tn insertion
R1	ATCGACCCAAGTACCGCCAC	Verification of *shrA* tn insertion
PA2444-F-NheI	GCCCCCGCTAGCAAGAAGGAGATATACCATGTTCAGCAAGCACGACCAGCTCCAGG	Construction of pMQ70-*shrA* and pMJT1-*shrA*
PA2444-R-HindIII	GCCCCCAAGCTTCTAGTGATGGTGATGGTGATGTCAATCAGCGTAGACCGGGAAGTGC	Construction of pMQ70-*shrA* and pET28b-PA2444cHis
PA2444-R-XbaI	GCCCCCTCTAGATCAATCAGCGTAGACCGGGAAGTGC	Construction of pMJT1-*shrA*
pMJT1-F	CTCTTCTCGCTAACCAAACC	Sequencing of pMJT1 derivatives
pMJT1-R	GTGGAATTGTGAGCGGAT	Sequencing of pMJT1 derivatives
PA2444-F-XbaI	GCCCCCTCTAGAAAGAAGGAGATATACCATGTTCAGCAAGCACGACCAGCTCCAGG	Construction of pET28b-PA2444cHis
pMQ70-F	GCGTCACACTTTGCTATGCCATAGC	Sequencing pMQ70 derivatives
pMQ70-R	CTACTGCCGCCAGGCAAATTCTGTTT	Sequencing pMQ70 derivatives
LuxAB inside	CTTTCAATTTCCGCTTTCAAGC	Identification of transposon insertion
LuxAB outside	CGATGGTGAGTTGTTCAAAATC	Identification of transposon insertion
Arb1	GGCCACGCGTCGACTAGTACNNNNNNNNNNGATAT	Identification of transposon insertion
Arb2	GGCCACGCGTCGACTAGTAC	Identification of transposon insertion
T7 promoter	TAATACGACTCACTATAGGG	Sequencing pET28b derivatives
T7 terminator	GCTAGTTATTGCTCAGCGG	Sequencing pET28b derivatives

### Complementation of *P. aeruginosa* mutants

For complementation of the *shrA* mutations, *shrA* was expressed under the control of the pBAD promoter in pMQ70 (Shanks et al., 2006) and in pMJT1 (Olsen et al., 1982). *shrA* was amplified using a Pfu DNA polymerase with primers PA2444-F-NheI and PA2444-R-HindIII (Table 3) for pMQ70-*shrA*, and primers PA2444-F-NheI and PA2444-R-XbaI for pMJT1-*shrA*. PCR products were cloned into the restriction sites of pMQ70 or pMJT1. The pMQ70-*shrA* and pMJT1-*shrA* plasmids were confirmed by DNA sequencing with the pMQ70-F/R primers or pMJT1-F/R primers (Table 3). pMQ70-*shrA* was transformed from the donor strain TG1/pMQ70-*shrA* into PA14 strains by conjugation using helper strain HB101/pRK2013 as described previously (Ueda and Wood, 2009). The plasmid pMJT1-*shrA* was transferred into the PA14 strains by electroporation (Choi et al., 2006).

### Colony morphology

Colony morphology was checked on Congo-red plates (10 g/L tryptone, 40 μg/mL Congo-red, 20 μg/mL Coomassie brilliant blue and 15 g/L agar) as described previously (Ueda and Wood, 2009) or with Congo-red plates supplemented with 30 mM potassium nitrate. Plates were incubated at 37°C or room temperature.

### Static biofilm assay

Biofilm formation was examined in 96-well polystyrene plates using crystal violet staining (Fletcher, 1977). Overnight cultures of *P. aeruginosa* were diluted to a turbidity of 0.05 at 600 nm with fresh LB medium, and then 200 μL of diluted bacterial culture was incubated in 96-well polystyrene plates for 4, 8, 24, and 50 h. Six wells were used for each strain, and at least two independent cultures were used for each experiment.

### Flow-cell biofilm assay

The flow cell experiments were performed as previously described (Ma et al., 2011) with modifications. At 37°C, the flow cells were inoculated with cultures in 5% LB medium at an initial turbidity at 600 nm of 0.05 and at a flow rate of 10 mL/h for 2 h, then fresh 5% LB medium was added at 10 mL/h for 3 days. After 72 h of incubation, biofilms were stained with SYTO9 for 20 min in the dark. Biofilm images from nine random positions were visualized with IMARIS confocal software (Bitplane, Zurich, Switzerland) and analyzed by COMSTAT confocal software (Heydorn et al., 2000).

### Motility assay

Swimming motility was examined using 0.3% agar plates with 1% tryptone and 0.25% NaCl as described previously (Sperandio et al., 2002), and swarming motility was examined with BM-2 plates (62 mM potassium phosphate, 2 mM MgSO_4_, 10 μM FeSO_4_, 0.1% casamino acid, 0.4% glucose, and 0.5% Bacto agar) (Overhage et al., 2008). Motility was measured after 24 h. Three plates were tested for each culture, and two independent cultures were used. The *flgK* (Liberati et al., 2006) and *rhlR* (Liberati et al., 2006) mutants were used as a negative controls for swimming and swarming, respectively.

### EPS and pyocyanin assay

EPS production was quantified by two methods. The first method (Ueda and Wood, 2009) was based on the amount of Congo-red that binds to the EPS in T-broth. PA14 *pelA* mutant (Liberati et al., 2006) was used as the negative control. Due to aggregative phenotype of the *shrA* mutant, cell pellets for all strains were sonicated at 3 W for 10 s three times to dissociate the cells as described previously (Ueda and Wood, 2009). Cell viability after sonication was tested by CFU counting using the drop plating method to make sure the mild sonication method did not cause cell lysis. The second method used anthrone-H_2_SO_4_ (Zhang et al., 2008) for quantification of the glucose equivalents in EPS. Normalized pyocyanin levels were assayed spectrophotometrically after overnight growth in LB medium as described previously after chloroform extraction (Wood and Wood, 2016).

### Total RNA isolation and microarray analysis

The *P. aeruginosa* genome array (Affymetrix, P/N 510596) was used to investigate differential gene expression in biofilm cells between PA14 and the *shrA* mutant. Biofilm cells were harvested from 10 g of glass wool (Ren et al., 2004a) after incubation for 7 h in LB with shaking at 250 rpm, and RNA was extracted with the RNeasy Mini Kit (Qiagen, Valencia, CA) using a bead beater (Biospec, Bartlesville, OK) (Ren et al., 2004a) with RNA*later* buffer (Applied Biosystems, Foster City, CA) to stabilize the RNA. cDNA synthesis, fragmentation, hybridizations, and data analysis were as described previously (González Barrios et al., 2006). For each binary microarray comparison of differential genes expression, if the gene with the larger transcription rate did not have a consistent transcription rate based on the 13 probe pairs (*P* <0.05), these genes were discarded. A gene was considered differentially expressed when the *P*-value for comparing two chips was lower than 0.05 (to assure that the change in gene expression was statistically significant and that false positives arise <5%) and when the expression ratio was higher than the standard deviation for the whole microarrays (1.8 for PA14 wild-type and *shrA mutant*) (Ren et al., 2004b). The microarray raw data are deposited at the Gene Expression Omnibus (GSE29879) of the National Center for Biotechnology Information; all data are MIAME compliant and have been deposited in a MIAME compliant database as detailed on the Functional Genomics Data Society website http://fged.org/projects/miame/.

### Genetic suppressor screening

To isolate the suppressive loci for RSCV formation due to the *shrA* mutation, a double mutant library was generated using the Tn*5-luxAB* transposon with the *shrA* strain as described previously (Ramsey and Whiteley, 2004). Screening cells with mutations in addition to *shrA* was performed in two steps as described previously (Ueda and Wood, 2009) with the first step used to discard cells with the aggregative phenotype and the second step to pick *P. aeruginosa* double mutants with smooth surfaces (*shrA* was red and wrinkled). The insertion position of Tn*5-luxAB* transposon was determined by two-step PCR as described previously (Ramsey and Whiteley, 2004) with primers LuxAB inside and Arb1 for the first round PCR and LuxAB outside and Arb2 for the second round PCR (Table 3). The PCR product was sequenced using primer LuxAB outside and a BigDye Terminator Cycle Sequencing kit (Applied Biosystems, Foster City, CA).

### c-di-GMP assay

c-di-GMP was quantified using HPLC as described previously (Ueda and Wood, 2009) with slight modifications. Strains were grown in 1 L LB medium for 16 h at 250 rpm. HPLC was conducted using C18 reverse-phase column (150^*^3.9 mm, 4 μm, Nova-Pak, Waters) at a flow rate of 1 ml/min. Solvent A was 0.15 M TEAA buffer (pH 5.0). Solvent B was acetonitrile. The gradient was as follows: *t* = 0, 0% solvent B; *t* = 35 min, 12% solvent B; *t* = 36 min, 80% solvent B; *t* = 41 min, 80% solvent B; *t* = 42 min, 0% solvent B; *t* = 55 min, 0% solvent B. Each sample had a running time of 55 min. A photodiode array detector (Waters, Milford, MA) was used to detect nucleotides at 254 nm after the HPLC separation step. Synthetic c-di-GMP (BIOLOG Life Science Institute, Bremen, Germany) was used as a standard. The peak corresponding to c-di-GMP from the extract of the *shrA* mutant was verified by co-elution with standard c-di-GMP. This experiment was performed with two independent cultures.

### Plasmid construction of pET28b-PA2444cHis and purification of recombinant ShrA-cHis

*shrA* was amplified with Pfu DNA polymerase using primers PA2444-F-XbaI and PA2444-R-HindIII (Table 3). The PCR product was digested with XbaI and HindIII and was cloned into the XbaI and HindIII sites of the pET28b vector. The resulting plasmid, pET28b-PA2444cHis has *shrA* fused to a 6 × His tag at the C-terminus (ShrA-cHis) and under control of the T7 promoter. The pET28b-PA2444cHis plasmid was confirmed by DNA sequencing with the T7 promoter and T7 terminator primers (Table 3). Production of ShrA-cHis was induced in *E. coli* BL21(DE3) cells with 1 mM IPTG at a turbidity of 1.0 at 600 nm overnight at room temperature. Cells were resuspended in 20 mL lysis buffer (50 mM potassium phosphate buffer, pH 7.6, 400 mM NaCl, 1 mM dithiothreitol (DTT), and 50 μM PLP) and disrupted twice by a French Press (Thermo Electron Corporation, Waltham, MA). ShrA-cHis was purified using a Ni-NTA resin (Qiagen, Valencia, CA) as described by the manufacturer's protocol. Purified ShrA-cHis was dialyzed against buffer (50 mM potassium phosphate buffer, pH 7.6, 50 mM NaCl, 8% glycerol, and 1 mM DTT) for three times at 4°C overnight to remove free PLP. Then the protein was concentrated using a 10 kDa cut-off centrifugal concentrator (Millipore, Billerica, MA), and the protein concentration was measured by using a Pierce BCA assay kit (Pierce, Rockford, IL).

### ShrA enzyme activity

The forward and backward SHMT activities were determined using an HPLC-based fluorometric assay using purified recombinant protein. For the backward reaction toward the production of glycine and (6*S*)-5,10-CH_2_-THF (Figure 1A), the reaction mixture was prepared as described previously (Chaturvedi and Bhakuni, 2003) with slight modification. Briefly, 100 μL of assay mixture contained 50 mM sodium phosphate buffer, pH 7.6, 4 or 6 mM *L*-Ser, 2 or 3 mM (6*R,S*)-THF, 2 mM DTT, 1 mM EDTA, 50 μM PLP and approximately 0.5 μM of purified ShrA. After incubation at 37°C for 20 min, the reaction was stopped by boiling for 10 min and centrifuged at 13,000 rpm for 20 min to remove protein. The amount of Ser and Gly was quantified using the amino acid analysis by HPLC equipped with a fluorescence detector as described previously in (Wu and Meininger, 2008). Generally, 50 μl supernatant was treated with 50 μl of 1.5 M HClO_4_ for 3 min, and then neutralized using 25 μl of 2 M K_2_CO_3_ and 1.125 ml H_2_O. The sample was vortexed and centrifuged to obtain the supernatant for HPLC analysis. For HPLC, the sample was mixed with o-phthaldialdehyde (OPA) reagent solution for 1 min in a reaction loop and immediately delivered into the C_18_ column without delay. Amino acids react with OPA in the presence of β-mercaptoethanol to form highly fluorescent products.

For the forward reaction toward the production of serine and (6*S*)-THF (Figure 1A), the reaction mixture was prepared as described previously (Wei and Roje, 2011) with slight modifications. Briefly, (6*R,S*)-5,10-CH_2_-THF was obtained by incubating (6*R,S*)-THF with 13 mM formaldehyde in 50 mM sodium phosphate buffer (pH 7.6) with 1 mM DETA. For the reaction, 100 μL assay mixture contained 50 mM sodium phosphate buffer, pH 7.6, 20 or 30 mM Gly, 2 or 3 mM (6*R,S*)-5,10-CH_2_-THF, 7.8 mM formaldehyde, 2 mM DTT, 1 mM EDTA, 50 μM PLP and approximately 0.5 μM of purified ShrA. The excess amount of formaldehyde ensures that the product (6*S*)-THF was immediately recycled back the substrate (6*S*)-5,10-CH_2_-THF, thereby the substrate (6*R,S*)-5,10-CH_2_-THF concentration was constant throughout the reaction. After incubating at 37°C for 20 min, the reaction was stopped by boiling for 10 min and centrifuged at 13,000 rpm for 20 min to remove protein.

### Oligomerization state of ShrA by cross-linking using glutaraldehyde

ShrA cross-linking was performed using a mild approach which employs the glutaraldehyde vapor as described previously (Fadouloglou et al., 2008). Briefly, ShrA protein was prepared as 0.5 mg/mL in 50 mM sodium phosphate buffer (pH 7.5) with 50 mM NaCl and 1 mM DTT. The hanging drop protein solution in a cover slip was incubated with the vapor from the 25% glutaraldehyde solution in a sealed well for various time points, and the size of the cross-linked products was examined by SDS-PAGE.

### PLP content for ShrA

The amount of PLP bound to the enzyme was determined for the recombinant enzyme as described previously (Ulevitch and Kallen, 1977) after extensive dialysis of the purified recombinant enzyme. Briefly, PLP will react with l-cysteine to form thiazolidine, and the amount of thiazolidine produced was determined from the absorbance at 335 nm.

### Intracellular NADH and NAD^+^

Overnight cultures were diluted into fresh LB medium with an initial turbidity at 600 nm of 0.005. The culture (100 μL) was spread on LB plates and incubated at 37°C for 24 h. Cells on plates were collected by rapidly resuspending in 5 mL of LB and centrifuging. Extraction of NAD^+^ and NADH was carried out using an acid and alkaline extraction method (Price-Whelan et al., 2007), and the NAD^+^ and NADH concentrations were quantified using an enzyme cycling assay with alcohol dehydrogenase (Price-Whelan et al., 2007) with slight modifications. Room temperature was used for the cycling assay conditions, and NADH and NAD^+^ standard samples (1~10 μM) were used for calibration. Three independent cultures were used for each strain.

## Author contributions

TW conceived the project. MP, LS, SS, and TG conducted the experiments. MP and TW wrote the manuscript.

### Conflict of interest statement

The authors declare that the research was conducted in the absence of any commercial or financial relationships that could be construed as a potential conflict of interest.

## References

[B1] Arai H. Igarashi Y. Kodama T. (1995). Expression of the *nir* and *nor* genes for denitrification of *Pseudomonas aeruginosa* requires a novel CRP/FNR-related transcriptional regulator, DNR, in addition to ANR. FEBS Lett. 371, 73–76. 10.1016/0014-5793(95)00885-D 7664887

[B2] Attila C. Ueda A. Wood T. K. (2008). PA2663 (PpyR) increases biofilm formation in *Pseudomonas aeruginosa* PAO1 through the *psl* operon and stimulates virulence and quorum-sensing phenotypes. Appl. Microbiol. Biotechnol. 78, 293–307. 10.1007/s00253-007-1308-y 18157527

[B3] Bantinaki E. Kassen R. Knight C. G. Robinson Z. Spiers A. J. Rainey P. B. (2007). Adaptive divergence in experimental populations of *Pseudomonas fluorescens*. III. Mutational origins of wrinkly spreader diversity. Genetics 176, 441–453. 10.1534/genetics.106.069906 17339222 PMC1893022

[B4] Berg J. M. Tymoczko J. L. Stryer L. (2002). Biochemistry. New York, NY: W. H. Freeman and Company.

[B5] Bhatt A. N. Bhakuni V. Kumar A. Khan M. Y. Siddiqi M. I. (2010). Alkaline pH-dependent differential unfolding characteristics of mesophilic and thermophilic homologs of dimeric serine hydroxymethyltransferase. Biochim. Biophys. Acta 1804, 1294–1300. 10.1016/j.bbapap.2010.01.023 20152942

[B6] Bodelón G. Montes-García V. López-Puente V. Hill E. H. Hamon C. Sanz-Ortiz M. N.. (2016). Detection and imaging of quorum sensing in *Pseudomonas aeruginosa* biofilm communities by surface-enhanced resonance Raman scattering. Nat. Materials 15, 1203–1211. 10.1038/nmat4720 27500808 PMC5082732

[B7] Chaturvedi S. Bhakuni V. (2003). Unusual structural, functional, and stability properties of serine hydroxymethyltransferase from *Mycobacterium tuberculosis*. J. Biol. Chem. 278, 40793–40805. 10.1074/jbc.M306192200 12913008

[B8] Choi K. H. Kumar A. Schweizer H. P. (2006). A 10-min method for preparation of highly electrocompetent *Pseudomonas aeruginosa* cells: application for DNA fragment transfer between chromosomes and plasmid transformation. J. Microbiol. Methods 64, 391–397. 10.1016/j.mimet.2005.06.001 15987659

[B9] Cornelis P. Matthijs S. Van Oeffelen L. (2009). Iron uptake regulation in *Pseudomonas aeruginosa*. Biometals 22, 15–22. 10.1007/s10534-008-9193-0 19130263

[B10] Dev I. K. Harvey R. J. (1984a). Role of methionine in the regulation of the synthesis of serine hydroxymethyltransferase in *Escherichia coli*. J. Biol. Chem. 259, 8402–8406. 6376505

[B11] Dev I. K. Harvey R. J. (1984b). Regulation of synthesis of serine hydroxymethyltransferase in chemostat cultures of *Escherichia coli*. J. Biol. Chem. 259, 8394–8401. 6376504

[B12] Dietrich L. E. Teal T. K. Price-Whelan A. Newman D. K. (2008). Redox-active antibiotics control gene expression and community behavior in divergent bacteria. Science 321, 1203–1206. 10.1126/science.1160619 18755976 PMC2745639

[B13] Ditta G. Stanfield S. Corbin D. Helinski D. R. (1980). Broad host range DNA cloning system for gram-negative bacteria: construction of a gene bank of *Rhizobium meliloti*. Proc. Natl. Acad. Sci. U.S.A. 77, 7347–7351. 10.1073/pnas.77.12.7347 7012838 PMC350500

[B14] Drenkard E. Ausubel F. M. (2002). *Pseudomonas* biofilm formation and antibiotic resistance are linked to phenotypic variation. Nature 416, 740–743. 10.1038/416740a 11961556

[B15] Fadouloglou V. E. Kokkinidis M. Glykos N. M. (2008). Determination of protein oligomerization state: two approaches based on glutaraldehyde crosslinking. Anal. Biochem. 373, 404–406. 10.1016/j.ab.2007.10.027 18023408

[B16] Figurski D. H. Helinski D. R. (1979). Replication of an origin-containing derivative of plasmid RK2 dependent on a plasmid function provided *in trans*. Proc. Natl. Acad. Sci. U.S.A. 76, 1648–1652. 10.1073/pnas.76.4.1648 377280 PMC383447

[B17] Fletcher M. (1977). The effects of culture concentration and age, time, and temperature on bacterial attachment to polystyrene. Can. J. Microbiol. 23, 1–6. 10.1139/m77-001

[B18] Florio R. di Salvo M. L. Vivoli M. Contestabile R. (2010). Serine hydroxymethyltransferase: a model enzyme for mechanistic, structural, and evolutionary studies. Biochim Biophys Acta 1814, 1489–1496. 10.1016/j.bbapap.2010.10.010 21059411

[B19] Giardina G. Rinaldo S. Johnson K. A. Di Matteo A. Brunori M. Cutruzzolà F. (2008). NO sensing in *Pseudomonas aeruginosa*: structure of the transcriptional regulator DNR. J. Mol. Biol. 378, 1002–1015. 10.1016/j.jmb.2008.03.013 18420222

[B20] Gjødsbøl K. Christensen J. J. Karlsmark T. Jørgensen B. Klein B. M. Krogfelt K. A. (2006). Multiple bacterial species reside in chronic wounds: a longitudinal study. Int. Wound J. 3, 225–231. 10.1111/j.1742-481X.2006.00159.x 16984578 PMC7951738

[B21] González Barrios A. F. Zuo R. Hashimoto Y. Yang L. Bentley W. E. Wood T. K. (2006). Autoinducer 2 controls biofilm formation in *Escherichia coli* through a novel motility quorum-sensing regulator (MqsR, B3022). J. Bacteriol. 188, 305–316. 10.1128/JB.188.1.305-316.2006 16352847 PMC1317603

[B22] Häussler S. (2010). Multicellular signalling and growth of *Pseudomonas aeruginosa*. Int. J. Med. Microbiol. 300, 544–548. 10.1016/j.ijmm.2010.08.006 20947425

[B23] Hengge R. (2009). Principles of c-di-GMP signalling in bacteria. Nat. Rev. Microbiol. 7, 263–273. 10.1038/nrmicro2109 19287449

[B24] Heydorn A. Nielsen A. T. Hentzer M. Sternberg C. Givskov M. Ersbϕll B. K.. (2000). Quantification of biofilm structures by the novel computer program COMSTAT. Microbiology 146, 2395–2407. 10.1099/00221287-146-10-2395 11021916

[B25] Hickman J. W. Tifrea D. F. Harwood C. S. (2005). A chemosensory system that regulates biofilm formation through modulation of cyclic diguanylate levels. Proc. Natl. Acad. Sci. U.S.A. 102, 14422–14427. 10.1073/pnas.0507170102 16186483 PMC1234902

[B26] Köhler T. Curty L. K. Barja F. van Delden C. Pechère J. C. (2000). Swarming of *Pseudomonas aeruginosa* is dependent on cell-to-cell signaling and requires flagella and pili. J. Bacteriol. 182, 5990–5996. 10.1128/JB.182.21.5990-5996.2000 11029417 PMC94731

[B27] Kulasakara H. Lee V. Brencic A. Liberati N. Urbach J. Miyata S.. (2006). Analysis of *Pseudomonas aeruginosa* diguanylate cyclases and phosphodiesterases reveals a role for bis-(3′-5′)-cyclic-GMP in virulence. Proc. Natl. Acad. Sci. U.S.A. 103, 2839–2844. 10.1073/pnas.0511090103 16477007 PMC1413825

[B28] Liberati N. T. Urbach J. M. Miyata S. Lee D. G. Drenkard E. Wu G.. (2006). An ordered, nonredundant library of *Pseudomonas aeruginosa* strain PA14 transposon insertion mutants. Proc. Natl. Acad. Sci. U.S.A. 103, 2833–2838. 10.1073/pnas.0511100103 16477005 PMC1413827

[B29] Lo Y.-L. Shen L. Chang C.-H. Bhuwan M. Chiu C.-H. Chang H.-Y. (2016). Regulation of motility and phenazine pigment production by flia is cyclic-di-GMP dependent in *Pseudomonas aeruginosa* PAO1. PLoS ONE 11:e0155397. 10.1371/journal.pone.0155397 27175902 PMC4866697

[B30] Lundgren B. R. Thornton W. Dornan M. H. Villegas-Peñaranda L. R. Boddy C. N. Nomura C. T. (2013). Gene PA2449 Is Essential for Glycine Metabolism and Pyocyanin Biosynthesis in *Pseudomonas aeruginosa* PAO1. J. Bacteriol. 195, 2087–2100. 10.1128/JB.02205-12 23457254 PMC3624589

[B31] Ma Q. Yang Z. Pu M. Peti W. Wood T. K. (2011). Engineering a novel c-di-GMP-binding protein for biofilm dispersal. Environ. Microbiol. 13, 631–642. 10.1111/j.1462-2920.2010.02368.x 21059164 PMC3057496

[B32] Macé C. Seyer D. Chemani C. Cosette P. Di-Martino P. Guery B.. (2008). Identification of biofilm-associated cluster (*bac*) in *Pseudomonas aeruginosa* involved in biofilm formation and virulence. PLoS ONE 3:e3897. 10.1371/journal.pone.0003897 19065261 PMC2587700

[B33] Malone J. G. Jaeger T. Spangler C. Ritz D. Spang A. Arrieumerlou C.. (2010). YfiBNR mediates cyclic di-GMP dependent small colony variant formation and persistence in *Pseudomonas aeruginosa*. PLoS Pathog. 6:e1000804. 10.1371/journal.ppat.1000804 20300602 PMC2837407

[B34] Meissner A. Wild V. Simm R. Rohde M. Erck C. Bredenbruch F.. (2007). *Pseudomonas aeruginosa cupA*-encoded fimbriae expression is regulated by a GGDEF and EAL domain-dependent modulation of the intracellular level of cyclic diguanylate. Environ. Microbiol. 9, 2475–2485. 10.1111/j.1462-2920.2007.01366.x 17803773

[B35] Minami M. Ando T. Hashikawa S. N. Torii K. Hasegawa T. Israel D. A.. (2004). Effect of glycine on *Helicobacter pylori in vitro*. Antimicrob. Agents Chemother. 48, 3782–3788. 10.1128/AAC.48.10.3782-3788.2004 15388434 PMC521915

[B36] Ochsner U. A. Vasil M. L. (1996). Gene repression by the ferric uptake regulator in *Pseudomonas aeruginosa*: cycle selection of iron-regulated genes. Proc. Natl. Acad. Sci. U.S.A. 93, 4409–4414. 8633080 10.1073/pnas.93.9.4409 PMC39551

[B37] Olsen R. H. DeBusscher G. McCombie W. R. (1982). Development of broad-host-range vectors and gene banks: self-cloning of the *Pseudomonas aeruginosa* PAO chromosome. J. Bacteriol. 150, 60–69. 6277872 10.1128/jb.150.1.60-69.1982 PMC220082

[B38] O'Toole G. A. Kolter R. (1998). Flagellar and twitching motility are necessary for *Pseudomonas aeruginosa* biofilm development. Mol. Microbiol. 30, 295–304. 10.1046/j.1365-2958.1998.01062.x 9791175

[B39] Overhage J. Bains M. Brazas M. D. Hancock R. E. W. (2008). Swarming of *Pseudomonas aeruginosa* is a complex adaptation leading to increased production of virulence factors and antibiotic resistance. J. Bacteriol. 190, 2671–2679. 10.1128/JB.01659-07 18245294 PMC2293252

[B40] Price-Whelan A. Dietrich L. E. Newman D. K. (2007). Pyocyanin alters redox homeostasis and carbon flux through central metabolic pathways in *Pseudomonas aeruginosa* PA14. J. Bacteriol. 189, 6372–6381. 10.1128/JB.00505-07 17526704 PMC1951912

[B41] Pu M. Wood T. K. (2010). Tyrosine phosphatase TpbA controls rugose colony formation in *Pseudomonas aeruginosa* by dephosphorylating diguanylate cyclase TpbB. Biochem. Biophys. Res. Commun. 402, 351–355. 10.1016/j.bbrc.2010.10.032 20946878 PMC2981663

[B42] Ramos I. Dietrich L. E. Price-Whelan A. Newman D. K. (2010). Phenazines affect biofilm formation by *Pseudomonas aeruginosa* in similar ways at various scales. Res. Microbiol. 161, 187–191. 10.1016/j.resmic.2010.01.003 20123017 PMC2886020

[B43] Ramsey M. M. Whiteley M. (2004). *Pseudomonas aeruginosa* attachment and biofilm development in dynamic environments. Mol. Microbiol. 53, 1075–1087. 10.1111/j.1365-2958.2004.04181.x 15306012

[B44] Rao N. A. Ambili M. Jala V. R. Subramanya H. S. Savithri H. S. (2003). Structure-function relationship in serine hydroxymethyltransferase. Biochim. Biophys. Acta 1647, 24–29. 10.1016/S1570-9639(03)00043-8 12686103

[B45] Ratomahenina R. Arthaud J. F. Galzy P. (1979). Inhibition by glycine of the growth of methylotrophic bacteria - some resistant mutants. Biotechnol. Lett. 1, 61–64. 10.1007/BF01398309

[B46] Ratomahenina R. Galzy P. (1981). Mutation modifying the serine pathway in methylotrophic bacteria. Folia Microbiol. (Praha) 26, 179–183. 10.1007/BF02927420 6792008

[B47] Ren D. Bedzyk L. A. Thomas S. M. Ye R. W. Wood T. K. (2004a). Gene expression in *Escherichia coli* biofilms. Appl. Microbiol. Biotechnol. 64, 515–524. 10.1007/s00253-003-1517-y 14727089

[B48] Ren D. Bedzyk L. A. Ye R. W. Thomas S. M. Wood T. K. (2004b). Differential gene expression shows natural brominated furanones interfere with the autoinducer-2 bacterial signalling system of *Escherichia coli*. Biotech. Bioeng. 88, 630–642. 10.1002/bit.20259 15470704

[B49] Ryder C. Byrd M. Wozniak D. J. (2007). Role of polysaccharides in *Pseudomonas aeruginosa* biofilm development. Curr. Opin. Microbiol. 10, 644–648. 10.1016/j.mib.2007.09.010 17981495 PMC2176169

[B50] Sambrook J. Fritsch E. F. Maniatis T. (1989). Molecular Cloning, A Laboratory Manual. Cold Spring Harbor, NY: Cold Spring Harbor Laboratory Press.

[B51] Sarwar Z. Lundgren B. R. Grassa M. T. Wang M. X. Gribble M. Moffat J. F. (2016). GcsR, a TyrR-like enhancer-binding protein, regulates expression of the glycine cleavage system in *Pseudomonas aeruginosa* PAO1. mSphere 1:e00020-16 10.1128/mSphere.00020-16 PMC4894688 27303730

[B52] Scarsdale J. N. Radaev S. Kazanina G. Schirch V. Wright H. T. (2000). Crystal structure at 2.4 Å resolution of *E. coli* serine hydroxymethyltransferase in complex with glycine substrate and 5-formyl tetrahydrofolate. J. Mol. Biol. 296, 155–168. 10.1006/jmbi.1999.3453 10656824

[B53] Schirch V. Szebenyi D. M. (2005). Serine hydroxymethyltransferase revisited. Curr. Opin. Chem. Biol. 9, 482–487. 10.1016/j.cbpa.2005.08.017 16125438

[B54] Schuster M. Lostroh C. P. Ogi T. Greenberg E. P. (2003). Identification, timing, and signal specificity of *Pseudomonas aeruginosa* quorum-controlled genes: a transcriptome analysis. J. Bacteriol. 185, 2066–2079. 10.1128/JB.185.7.2066-2079.2003 12644476 PMC151497

[B55] Shanks R. M. Q. Caiazza N. C. Hinsa S. M. Toutain C. M. O'Toole G. A. (2006). *Saccharomyces cerevisiae*-based molecular tool kit for manipulation of genes from gram-negative bacteria. Appl. Environ. Microbiol. 72, 5027–5036. 10.1128/AEM.00682-06 16820502 PMC1489352

[B56] Shoeman R. Redfield B. Coleman T. Greene R. C. Smith A. A. Brot N.. (1985). Regulation of methionine synthesis in *Escherichia coli*: effect of *metJ* gene product and S-adenosylmethionine on the expression of the *metF* gene. Proc. Natl. Acad. Sci. U.S.A. 82, 3601–3605. 10.1073/pnas.82.11.3601 16593564 PMC397833

[B57] Simm R. Morr M. Remminghorst U. Andersson M. Römling U. (2009). Quantitative determination of cyclic diguanosine monophosphate concentrations in nucleotide extracts of bacteria by matrix-assisted laser desorption/ionization-time-of-flight mass spectrometry. Anal. Biochem. 386, 53–58. 10.1016/j.ab.2008.12.013 19135022

[B58] Simon R. Priefer U. Pühler A. (1983). A broad host range mobilization system for *in vivo* genetic engineering: transposon mutagenesis in gram-negative bacteria. Nat. Biotechnol. 1, 784–791. 10.1038/nbt1183-784

[B59] Sperandio V. Torres A. G. Kaper J. B. (2002). Quorum sensing *Escherichia coli* regulators B and C (QseBC): a novel two-component regulatory system involved in the regulation of flagella and motility by quorum sensing in E. coli. Mol. Microbiol. 43, 809–821. 10.1046/j.1365-2958.2002.02803.x 11929534

[B60] Springer W. R. Koshland D. E. Jr. (1977). Identification of a protein methyltransferase as the *cheR* gene product in the bacterial sensing system. Proc. Natl. Acad. Sci. U.S.A. 74, 533–537. 10.1073/pnas.74.2.533 322131 PMC392324

[B61] Starkey M. Hickman J. H. Ma L. Zhang N. De Long S. Hinz A.. (2009). *Pseudomonas aeruginosa* rugose small-colony variants have adaptations that likely promote persistence in the cystic fibrosis lung. J. Bacteriol. 191, 3492–3503. 10.1128/JB.00119-09 19329647 PMC2681918

[B62] Stover P. Schirch V. (1990). Serine hydroxymethyltransferase catalyzes the hydrolysis of 5,10-methenyltetrahydrofolate to 5-formyltetrahydrofolate. J. Biol. Chem. 265, 14227–14233. 2201683

[B63] Ueda A. Attila C. Whiteley M. Wood T. K. (2009). Uracil influences quorum sensing and biofilm formation in *Pseudomonas aeruginosa* and fluorouracil is an antagonist. Microb. Biotechnol. 2, 62–74. 10.1111/j.1751-7915.2008.00060.x 21261882 PMC3815422

[B64] Ueda A. Wood T. K. (2009). Connecting quorum sensing, c-di-GMP, pel polysaccharide, and biofilm formation in *Pseudomonas aeruginosa* through tyrosine phosphatase TpbA (PA3885). PLoS Pathog. 5:e1000483. 10.1371/journal.ppat.1000483 19543378 PMC2691606

[B65] Ulevitch R. J. Kallen R. G. (1977). Purification and characterization of pyridoxal 5′-phosphate dependent serine hydroxymethylase from lamb liver and its action upon beta-phenylserines. Biochemistry 16, 5342–5350. 10.1021/bi00643a027 921936

[B66] Wei Z. Roje S. (2011). A high-performance liquid chromatography-based fluorometric method for assaying serine hydroxymethyltransferase toward serine formation. Anal. Biochem. 409, 156–158. 10.1016/j.ab.2010.10.004 20946865

[B67] Williams D. R. Rowe J. J. Romero P. Eagon R. G. (1978). Denitrifying *Pseudomonas aeruginosa*: some parameters of growth and active transport. Appl. Environ. Microbiol. 36, 257–263. 100056 10.1128/aem.36.2.257-263.1978 PMC291211

[B68] Wood T. L. Wood T. K. (2016). The HigB/HigA toxin/antitoxin system of *Pseudomonas aeruginosa* influences the virulence factors pyochelin, pyocyanin, and biofilm formation. Microbiologyopen 5, 499–511. 10.1002/mbo3.346 26987441 PMC4906001

[B69] Wu G. Meininger C. J. (2008). Analysis of citrulline, arginine, and methylarginines using high-performance liquid chromatography. Meth. Enzymol. 440, 177–189. 10.1016/S0076-6879(07)00810-5 18423217

[B70] Zhang X. S. García-Contreras R. Wood T. K. (2008). *Escherichia coli* transcription factor YncC (McbR) regulates colanic acid and biofilm formation by repressing expression of periplasmic protein YbiM (McbA). ISME J. 2, 615–631. 10.1038/ismej.2008.24 18309357

