# Supplementary material for: Serine Hydroxymethyltransferase ShrA (PA2444) Controls Rugose Small-Colony Variant Formation in Pseudomonas aeruginosa
Source: Front Microbiol. 2018 Feb 27;9:315. doi: 10.3389/fmicb.2018.00315 (PMC5835335; doi:10.3389/fmicb.2018.00315)
Supplement: Supplementary file 1 [file DataSheet1.PDF]

SUPPORTING INFORMATION

Serine Hydroxymethyltransferase ShrA (PA2444) Controls Rugose Small-Colony Variant Formation in *Pseudomonas aeruginosa*

Mingming Pu, Lili Sheng, Sooyeon Song, Ting Gong, and Thomas K. Wood*****

Department of Chemical Engineering, Pennsylvania State University,

University Park, Pennsylvania, 16802-4400, USA

*For correspondence. E-mail twood@engr.psu.edu

Tel.(+)1 814-863-4811; Fax (1) 814-865-7846

Table S1. Partial list of repressed genes in biofilm cells in LB medium after 7 h at 37°C for the *shrA* mutant versus wild-type PA14. Genes in the same cluster are listed together. Raw data for the DNA microarray are available using GEO series accession number GSE 29879. Note that the transcript level for *shrA* is repressed but not eliminated since a transposon mutant was used, not a gene knockout.

PAO1 ID	PA14 ID	Gene Name	Fold change	Description
Denitrification				
PA0509	PA14_06650	*nirN*	-2.5	probable c-type cytochrome
PA0510	PA14_06660		-4.6	probable uroporphyrin-III c-methyltransferase
PA0511	PA14_06670	*nirJ*	-3.7	heme d1 biosynthesis protein NirJ
PA0512	PA14_06680		-4.3	conserved hypothetical protein
PA0513	PA14_06690		-4.6	probable transcriptional regulator
PA0514	PA14_06700	*nirL*	-4.0	heme d1 biosynthesis protein NirL
PA0515	PA14_06710		-5.7	probable transcriptional regulator
PA0516	PA14_06720	*nirF*	-4.9	heme d1 biosynthesis protein NirF
PA0517	PA14_06730	*nirC*	-8.0	probable c-type cytochrome precursor
PA0518	PA14_06740	*nirM*	-7.5	cytochrome c-551 precursor
PA0519	PA14_06750	*nirS*	-6.5	nitrite reductase precursor
PA0520	PA14_06770	*nirQ*	-2.6	regulatory protein NirQ
PA0521	PA14_06790		-4.3	probable cytochrome c oxidase subunit
PA0522	PA14_06800		-2.3	hypothetical protein
PA0523	PA14_06810	*norC*	-21.1	nitric-oxide reductase subunit C
PA0524	PA14_06830	*norB*	-24.3	nitric-oxide reductase subunit B
PA0525	PA14_06840	*norD*	-11.3	probable dinitrification protein NorD
PA0526	PA14_06860		-3.0	hypothetical protein
PA3392	PA14_20200	*nosZ*	-5.7	nitrous-oxide reductase precursor
PA3395	PA14_20170	*nosY*	-1.9	nitrous-oxide reductase, nosY component
PA3396	PA14_20150	*nosL*	-5.3	putative nitrous-oxide reductase protein
Iron acquisition related, including siderophores				
PA2424	PA14_33280	*pvdL*	-4.0	probable non-ribosomal peptide synthetase
PA2425	PA14_33270	*pvdG*	-5.7	probable thioesterase
PA2426	PA14_33260	*pvdS*	-16.0	sigma factor PvdS, required for expression of other pvd genes
PA2427	PA14_33250		-6.5	hypothetical protein
PA2384	PA14_33830		-8.0	hypothetical protein
PA2385	PA14_33820	*pvdQ*	-7.0	probable acylase /FUNCTION
PA2386	PA14_33810	*pvdA*	-14.9	PL-ornithine N5-oxygenase, required for type I pyoverdine
PA2389	PA14_33770		-3.5	putative ABC export system
PA2390	PA14_33760		-2.3	probable ATP-binding/permease fusion ABC transporter
PA2391	PA14_33750	*opmQ*	-1.7	probable outer membrane protein
PA2392	PA14_33740	*pvdP*	-13.0	hypothetical protein, Pyoverdine synthesis
PA2393	PA14_33730		-14.9	probable dipeptidase precursor

PA2394	PA14_33720	*pvdN*	-7.0	probable aminotransferase
PA2395	PA14_33710	*pvdO*	-6.1	hypothetical protein
PA2396	PA14_33700	*pvdF*	-7.5	hypothetical protein
PA2397	PA14_33690	*pvdE*	-9.2	pyoverdine biosynthesis protein PvdE
PA2398	PA14_33680	*fpvA*	-5.3	ferripyoverdine receptor
PA2399	PA14_33650	*pvdD*	-3.5	pyoverdine synthetase D
PA2400	PA14_33630	*pvdJ*	-2.8	pyoverdine peptide synthetase
PA2401	PA14_33620		-4.9	probable non-ribosomal peptide synthetase
PA2402	PA14_33610		-7.5	probable non-ribosomal peptide synthetase
PA2403	PA14_33600		-4.0	hypothetical protein
PA2404	PA14_33590		-4.6	hypothetical protein
PA2405	PA14_33580		-5.3	hypothetical protein
PA2406	PA14_33570		-4.0	hypothetical protein
PA2407	PA14_33560		-2.8	probable adhesion protein
PA2408	PA14_33550		-3.7	probable ATP-binding component of ABC transporter
PA2409	PA14_33540		-3.5	probable permease of ABC transporter
PA2410	PA14_33530		-2.5	hypothetical protein
PA2411	PA14_33520		-6.5	probable thioesterase
PA2412	PA14_33510		-12.1	conserved hypothetical protein
PA2413	PA14_33500	*pvdH*	-4.9	probable class III aminotransferase, Pyoverdine synthesis
PA0672	PA14_55580	*hemO/pigA*	-8.6	heme oxygenase
PA0674	PA14_55560	*pigC*	-1.7	53% similar to ferripyoverdine receptor
PA0675	PA14_55550		-2.3	45% similar to positive regulator PvdS
PA4168	PA14_09970	*fpvB*	-4.3	second ferric pyoverdine receptor FpvB
PA4218	PA14_09380		-2.1	putative transpoter
PA4219	PA14_09370	*yfpB*	-1.7	putative membrane protein
PA4220	PA14_09350	*fptB*	-1.9	hypothetical protein
PA4221	PA14_09340	*fptA*	-2.0	FptA ferricpyochelin receptor
PA4222	PA14_09320	*pchI*	-2.0	probable ATP-binding component of ABC transporter
PA4223	PA14_09300	*pchH*	-1.9	probable ATP-binding component of ABC transporter
PA4224	PA14_09290	*pchG*	-1.7	pyochelin biosynthetic protein PchG
PA4225	PA14_09280	*pchF*	-2.3	pyochelin synthetase
PA4226	PA14_09270	*pchE*	-2.0	dihydroaeruginoic acid synthetase
PA4514	PA14_58570	*piuA*	-2.3	probable outer membrane receptor for iron transport
PA4708	PA14_62300	*phuT*	-3.2	53% similar to hemin binding protein HemT
PA4709	PA14_62330	*phuS*	-4.6	putative hemin degrading factor
PA4710	PA14_62350	*phuR*	-6.5	49% similar to heme receptor HutA
PA0931	PA14_52230	*pirA*	-2.8	ferric enterobactin receptor PirA
PA1911	PA14_39810		-2.5	upstream of ufrA, undefined ferric siderophore receptor
PA1912	PA14_39800		-2.8	71% similar to probable RNA polymerase sigma factor FecI
PA0470	PA14_06160	*fiuA*	-2.0	probable hydroxamate-type ferrisiderophore receptor
PA0471	PA14_06170	*fiuR*	-2.1	46% simialr to fecR gene product
PA0472	PA14_06180	*fiuI*	-2.3	67% similar to putative sigma factor FecI

PA4158	PA14_10180	*fepC*	-2.1	ferric enterobactin transport protein FepC
PA2686	PA14_29375	*pfeR*	-2.5	two-component response regulator PfeR
PA0197	PA14_02490	*tonB2*	-3.0	putative TonB protein
PA0198	PA14_02500	*exbB1*	-4.0	transport protein ExbB
PA0199	PA14_02510	*exbD1*	-3.2	transport protein ExbD
PA4467	PA14_57990		-4.9	hypothetical protein
PA4468	PA14_58000	*sodM/sodA*	-8.6	superoxide dismutase
PA4469	PA14_58010		-8.6	hypothetical protein
PA4470	PA14_58030	*fumC1*	-8.6	fumarate hydratase
PA4471	PA14_58040	*fagA*	-8.6	product of Fur-associated gene FagA
Redox related				
PA3444	PA14_19560	*ssuD*	-4.0	95% similar to putative FMNH2-dependent monooxygenase SsuD
PA3446	PA14_19530	*ssuE*	-3.5	putative NADH-dependent FMN reductase
PA3450	PA14_19490	*lsfA*	-2.1	probable thiol-specific antioxidant protein
PA0194	PA14_01840		-1.9	45% similar to taurine dioxygenase TauD
PA2594	PA14_30550		-2.5	putative periplasmic sulfonate-binding protein
PA2597	PA14_30500		-1.7	putative acyl-CoA dehydrogenase
PA2598	PA14_30490		-2.0	putative FMNH2-dependent monooxygenase
PA2599	PA14_30470		-2.0	putative periplasmic sulfonate-binding protein
PA2600	PA14_30460		-2.1	52% similar to putative FMNH2-dependent monooxygenase
PA3935	PA14_12970	*tauD*	-3.0	taurine dioxygenase
PA3936	PA14_12960	*tauC*	-3.2	probable permease of ABC taurine transporter
PA3937	PA14_12940	*tauB*	-2.8	probable ATP-binding component of ABC taurine transporter
PA3938	PA14_12920	*tauA*	-3.0	probable periplasmic taurine-binding protein precursor
shrA operon				
PA2442	PA14_33040	*gcvT2*	-4.0	glycine cleavage system protein T2
PA2443	PA14_33030	*sdaA*	-3.7	L-serine dehydratase
PA2444	PA14_33010	*shrA*	-11.3	serine hydroxymethyltransferase

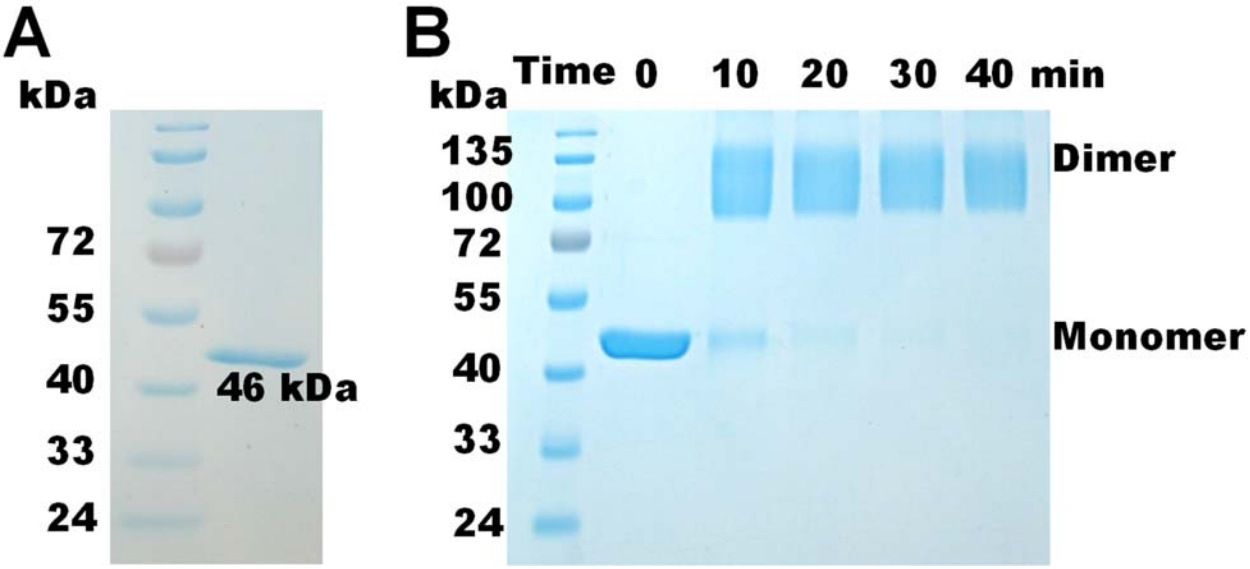

Fig. S1 Purification of ShrA and cross-linking assay. (**A**) SDS-PAGE of purified ShrA. (**B**) Cross-linking of ShrA by glutaraldehyde vapor at different time intervals.
